# The effect of linguistic comprehension instruction on generalized language and reading comprehension skills: A systematic review

**DOI:** 10.1002/cl2.1059

**Published:** 2019-11-07

**Authors:** Kristin Rogde, Åste M. Hagen, Monica Melby‐Lervåg, Arne Lervåg

**Affiliations:** ^1^ Nordic Institute for Studies in Innovation, Research and Education (NIFU) Oslo Norway; ^2^ Department of Special Needs Education University of Oslo Oslo Norway; ^3^ Department of Education University of Oslo Oslo Norway

## 1 PLAIN LANGUAGE SUMMARY

### 1.1 The review in brief

The linguistic comprehension programs included in this review display a small positive immediate effect on generalized outcomes of linguistic comprehension. The effect of the programs on generalized measures of reading comprehension is negligible. Few studies report follow‐up assessment of their participants.

### 1.2 What is this review about?

Children who begin school with proficient language skills are more likely to develop adequate reading comprehension abilities and achieve academic success than children who struggle with poor language skills in their early years. Individual language difficulties, environmental factors related to socioeconomic status (SES), and having the educational language as a second language are all considered risk factors for language and literacy failure.

Intervention programs have been designed with the aim of supporting at‐risk children's language skills. In these programs, the instructional methods typically include a strong focus on vocabulary instruction within the context of storytelling or text reading. Elements that directly activate narrative and grammatical development are often included.

**What is the aim of this review?**

This Campbell systematic review examines the effects of linguistic comprehension instruction on generalized measures of language and reading comprehension skills. The review summarizes evidence from 43 studies, including samples of both preschool and school‐aged participants.

### 1.3 What studies are included in this review?

This review included studies that evaluate the effects of linguistic comprehension interventions on generalized language and reading outcomes. A total of 43 studies were identified and included in the final analysis. The studies span the period 1992–2017. Randomized controlled trials (RCTs) and quasi‐experiments (QEs) with a control group and a pre–post design were included in the review.

### 1.4 What are the main findings of this review?

The effect of linguistic comprehension instruction on generalized outcomes of linguistic comprehension skills is small in studies of both the overall immediate and follow‐up effects. Analysis of differential language outcomes shows small effects on vocabulary and grammatical knowledge and moderate effects on narrative and listening comprehension.

Linguistic comprehension instruction has no immediate effects on generalized outcomes of reading comprehension. Only a few studies have reported follow‐up effects on reading comprehension skills, with divergent findings.

### 1.5 What do the findings of this review mean?

Linguistic comprehension instruction has the potential to increase children's general linguistic comprehension skills. However, there is variability in effects related to the type of outcome measure that is used to examine the effect of such instruction on linguistic comprehension skills.

One of the overall aims of linguistic comprehension intervention programs is to accelerate children's vocabulary development. Our results indicated that the type of intervention program included in this review might be insufficient to accelerate children's vocabulary development and, thus, to close the vocabulary gap among children.

Further, the absence of an immediate effect of intervention programs on reading comprehension outcomes indicates that linguistic comprehension instruction through the type of intervention program examined in this study does not transfer beyond what is learned to general types of text. Despite clear indications from longitudinal studies that linguistic comprehension plays a vital role in the development of reading comprehension, only a few intervention studies have produced immediate and follow‐up effects on generalized outcomes of reading comprehension. This indicates that preventing and remediating reading comprehension difficulties likely requires long‐term educational efforts.

Finally, it is likely that other outcome measures that are more closely aligned with the targeted intervention (use of targeted instructed words in the texts) would yield a different pattern of results. However, such tests were not included in this review.

### 1.6 How up‐to‐date is this review?

The review authors searched for studies up to October 2018.

## 2 EXECUTIVE SUMMARY/ABSTRACT

### 2.1 Background

Well‐developed vocabulary and language comprehension skills are not only critical in themselves but also fundamental to the development of adequate reading comprehension abilities and achieving academic success. Children with poor language skills, children from low socioeconomic areas, and second‐language learners are at risk for subsequent reading comprehension problems. Reading comprehension difficulties are relatively common in school‐aged children, and intervention programs have been designed to support children's linguistic comprehension skills.

### 2.2 Objectives

The primary objective of this review was to examine the extent to which linguistic comprehension instruction in educational settings is effective when measured by generalized outcomes of linguistic comprehension and reading comprehension.

### 2.3 Search methods

Specific electronic searches for literature dating back to 1986 were conducted in the following databases: Eric (Ovid), PsycINFO (Ovid), ISI Web of Science, Proquest Digital Dissertations, Linguistic and Language Behavior Abstracts (LLBA), Scopus Science Direct, Open Grey and Bielefeld Academic Search Engine (BASE). The search was limited to publications reported in English. The literature search also utilized citations, Google Scholar, prior meta‐analyses and key journals. In addition, authors in the field were contacted for unpublished or in‐press manuscripts.

### 2.4 Selection criteria

The review included RCTs and QEs with a pretest–posttest‐controlled design. It was imperative that the intervention programs were conducted in a preschool or later educational setting, up to the end of secondary school. Intervention programs implemented by parents or other persons in the child's home environment were not included in the review. Further, the sample of participants included both monolingual and second‐language learners, unselected typically achieving children, children with language delay/weaknesses, or children from low socioeconomic backgrounds. Samples of children with a special diagnosis, like autism, or other physical, mental, or sensory disabilities were not eligible for inclusion in the review. Moreover, studies had to report generalized outcomes of language and reading comprehension to be included in the review. Studies that only reported proximal outcomes designed by researchers to measure the direct effect of trained words were not included.

### 2.5 Data collection and analysis

Two electronic searches were conducted for this review. The first search was conducted in October 2016, followed by the same electronic search strategy in October 2018; 4,991 references for the original and 1,776 references for the follow‐up search were identified and screened for eligibility. Among these, 871 references for the original and 175 references for the follow‐up search were included for a full‐text screening procedure.

Analyses were conducted using the Comprehensive meta‐analysis program by Borenstein, Hedges, Higgins, and Rothstein (2014).

### 2.6 Results

Overall, 43 references met the inclusion criteria and were included in the review. Linguistic comprehension instruction showed small effects on generalized measures of vocabulary and grammar in favor of the treatment groups. Further, the effect of linguistic comprehension instruction on narrative and listening comprehension skills showed positive moderate effects in favor of the treatment groups. However, there was no clear evidence of effect of linguistic comprehension instruction on general reading comprehension outcomes from the type of trials included in this review.

### 2.7 Authors’ conclusions

The evidence indicated that the type of intervention program included in this review has the potential to increase children's general linguistic comprehension skills. However, these programs are probably not sufficiently effective to accelerate children's vocabulary knowledge and close the vocabulary gap among children. Programs with longer time frames and follow‐up assessments than what was included in this review must be developed in the future. Simultaneously, more information from RCTs is needed to ensure that no systematic differences between intervention groups affect the outcome.

## 3 BACKGROUND

### 3.1 The problem, condition, or issue

#### 3.1.1 Poor linguistic comprehension skills, prevalence, and associated problems

The ability to understand and express language in both its oral and written forms is a crucial aspect of human development. Language is vital to be able to communicate with others and is closely linked to both social and emotional functioning. Children with poor language skills may experience more problems related to social, emotional, and behavioral aspects relative to their peers (Norbury et al., 2016). Researchers indicate less engagement in conversational interactions, poorer discourse skills, and more communication misunderstandings among children with poor language skills as compared to their typical peers (Durkin & Conti‐Ramsden, 2007, 2010). Children with poor language skills are also considered to be at risk for poor academic achievement. Proficient language skills are fundamental to all higher‐level cognitive activities and set the stage for reading development and academic success (McNamara & Magliano, 2009).

Even though most children develop language naturally at a rapid pace, poor language skills in early childhood are not uncommon. An epidemiological study by Norbury et al. (2016) estimated the prevalence of language disorders of unknown origin to be approximately two children in every first‐year classroom (7.58%). Both genetic risk factors within the child (Puglisi, Hulme, Hamilton, & Snowling, 2017; Stromswold, 2001) combined with environmental factors related to the amount and quality of language exposure (Hoff, 2003) are likely to explain the large variations between children and why some children will be at a greater risk for developing poor language skills than others. In addition, substantial portions of children entering school across countries come from families in which a language other than the educational language is practiced. Even though researchers have indicated cognitive advantages of growing up multilingual, like executive control, (Bialystok & Viswanathan, 2009) benefits related to linguistic processing is not reported. In a meta‐analysis comparing first‐ and second‐language learners, Melby‐Lervåg and Lervåg (2014) found that second‐language learners displayed a large deficit in language comprehension (*d* = −1.12 in favor of first‐language learners). Thus, as a group, second‐language learners display poorer linguistic comprehension skills in the second language than their monolingual peers. Their challenges are particularly related to vocabulary acquisition in the second language, and there appears to be limited transfer from the first language to the second language (Melby‐Lervåg & Lervåg, 2011; Snow & Kim, 2006).

In school‐aged children, poor language skills may manifest themselves as reading comprehension problems. This becomes a problem when children reach fourth grade and are expected to begin reading to learn. In general, difficulties with reading comprehension are prevalent among students across countries. In the United States, 32% of students in the fourth grade and 24% of students in the eighth grade performed below the basic level on the National Assessment of Educational Progress (NAEP) reading test in 2017 (National Center for Educational Statistics [NCES], 2018). The proportion of children reading below the basic level is reported to be higher among children from families with low SES and from minority race/ethnicity groups, such as black and Hispanic children. In 2017, White fourth‐grade students outperformed their Black peers with 26 scaled scores, and Hispanic students with 23 scaled scores (NCES, 2018). Recent assessments on NAEP also showed that the average reading score for second‐language learners in eighth grade was 43 scaled scores lower than the average score for peers who are not second‐language learners (NCES, 2018). The situation of low‐level reading skills among students is similar in North America and several European countries (Organization for Economic Co‐operation and Development [OECD], 2010a, 2010b). As children who lack a strong foundation of linguistic and reading comprehension skills are more likely to experience academic difficulties and drop‐out from school, developing effective instructional practices is of the utmost importance to the field of education. The aim of this review was to improve our understanding of intervention studies targeting two core constructs: linguistic comprehension and reading comprehension.

*Linguistic comprehension* is defined as the process by which lexical (i.e., word) information, sentences, and discourses are interpreted (Gough & Tunmer, 1986). It refers to the ability to understand oral language, often assessed by tests of vocabulary or listening comprehension (Bornstein, Hahn, Putnick, & Suwalsky, 2014; Foorman, Herrera, Petscher, Mitchell, & Truckenmiller, 2015; Klem et al., 2015; Melby‐Lervåg & Lervåg, 2014). Vocabulary is a core component of linguistic comprehension. Vocabulary has typically been divided into either expressive and receptive vocabulary or depth and breadth vocabulary (Ouellette, 2006). However, several more recent studies, using latent variables, have shown that these are highly related constructs that are difficult to differentiate (e.g., Bornstein et al., 2014; Lervåg, Hulme, & Melby‐Lervåg, 2017). Although vocabulary is a core component in linguistic comprehension, skills such as syntax (the ability to understand and formulate sentences) and morphology (how words are formed), which build directly on vocabulary knowledge, are also often considered to be part of a broader linguistic comprehension construct (e.g., Klem et al., 2015).

*Reading comprehension* can be defined as the active extraction and construction of meaning from all kinds of texts (Snow, 2001). Linguistic comprehension is commonly understood as an important factor that underpins the development of reading comprehension beyond word‐level reading (Gough & Tunmer, 1986). In later grades, when decoding skills are fully mastered and the contribution of decoding skills to reading comprehension has lessened, linguistic comprehension and reading comprehension are almost isomorphic constructs (Lervåg et al., 2017; Muter, Hulme, Snowling, & Stevenson, 2004; Storch & Whitehurst, 2002).

The primary aim of this review was to provide an overview of studies on interventions targeting linguistic comprehension and their effects on measures of generalized linguistic comprehension skills. Because linguistic comprehension skills are understood to be a prerequisite for subsequent reading comprehension skills, the second aim of this review was to examine possible transfer effects from instruction to generalized reading comprehension outcomes. We know from earlier trials and prior reviews that children learn words that they have been directly instructed in. However, the evidence to which educational intervention programs can produce effects on generalized language and reading comprehension tests that are not targeted for specific intervention (distal effects) has been unclear. Moreover, it must be noted that the terms *generalized* linguistic comprehension and reading comprehension outcomes refer to tests that are not targeted for the specific intervention. This implies that it is the distal treatment effects that are of interest and that the outcomes are not inherent to treatment (e.g., standardized tests; see Cheung & Slavin, 2016).

By strengthening our knowledge of this subject, we can potentially obtain insights into how related deficits can be ameliorated. This information is critical in making policy decisions regarding whether such programs are suitable for implementation in early childhood education and later schooling. In addition, reviewing intervention studies may also provide a more refined understanding of the underlying causal mechanisms through which interventions are effective. This aspect is vital for providing a sound theoretical foundation for constructing better and more targeted intervention programs.

### 3.2 The intervention

#### 3.2.1 Participants

In general, intervention research that targets linguistic comprehension instruction typically targets groups of children who are at risk for language and reading comprehension difficulties. Samples of participants may represent classroom students from low socioeconomic areas, children with poor language skills who are selected based on screening tests for language proficiency, and samples with participants who have their educational language as a second language.

### 3.3 Content

The content of the intervention programs reviewed in this paper involves instruction in linguistic comprehension skills (e.g., vocabulary, grammar, and narrative skills). The intervention programs of interest aim, at an overall level, to provide children or students with rich exposures to language learning situations. The overall aim was to obtain the effects of linguistic instruction on generalized outcomes of linguistic comprehension and reading comprehension.

Vocabulary instruction is the main building block in the design of linguistic comprehension instruction programs. Vocabulary knowledge serves as a proxy for the development of spoken language skills and plays a crucial role in the understanding of texts (Anderson & Freebody, 1981; Graves, 1986). Therefore, the focus on vocabulary instruction is highly valued in experimental studies among both preschool and school‐aged children.

Recognized theorists have identified principles for effective word teaching that are typically included as instructional features in the type of studies examined in this review: the instruction must provide definitional and contextual information and repeated exposures as well as facilitate active processing (e.g., McKeown & Beck, 2014; Stahl & Fairbanks, 1986). However, learning a number of word meanings does not necessarily provide children with the competence to acquire the knowledge of new words independently. Therefore, intervention studies typically provide direct word instruction as an embedded feature within a broader comprehensive program. Then, the aim is to obtain effects on generalized outcomes of linguistic comprehension and reading comprehension (both of which are the focus of this review).

A commonly employed strategy to teach children words in intervention studies has been to provide children with direct instruction in word meanings through storybook reading. This direct vocabulary instruction has been practiced in various ways. One instructional approach is to provide children with brief explanations of word meanings during reading. This embedded vocabulary instruction targets the breadth of their vocabulary knowledge and has the benefit of being time‐efficient, as it allows for the instruction of numerous word meanings during a training session (Coyne, McCoach, Loftus, Zipoli, & Kapp, 2009). Another instructional approach is to provide children with a rich instruction of words following storybook reading (Beck & McKeown, 2007). This includes providing multiple explanations and examples related to multiple contexts and letting children actively engage in the explanations and discussions of word meanings. This technique is expected to foster a child's depth of vocabulary knowledge, in contrast to increasing the number of word meanings that a child knows (Beck & McKeown, 2007; Coyne et al., 2009). In addition to the quantity of instruction and direct word instruction, experimental programs are typically designed based on principals for instructional quality of interactions and extended talk during activities (Snow, Tabors, & Dickinson, 2001). Selected topics and instruction of words are typically used as a gateway for discussions (Weizman & Snow, 2001). Shared book reading is a common recommended activity to support young children's language skills (Dickinson & Tabors, 2001), and a commonly used activity in experimental studies. Shared book reading implemented using the methodology known as dialogic reading (Whitehurst et al., 1994) provides an opportunity for the instruction of words that are presented in text along with opportunities to focus the instruction on active listening skills and building narrative competencies. Similarly, intervention programs for school‐aged children typically value vocabulary instruction that contains explicit explanations within the context of both discussions and text reading (Lawrence, Crosson, Paré‐Blagoev, & Snow, 2015) and utilizes instructional features to increase students’ word consciousness by building morphological awareness (Brinchmann, Hjetland, & Lyster, 2015; Lesaux, Kieffer, Kelley, & Harris, 2014).

In closing, even though the principals of vocabulary instruction and principals for instruction are often aligned across studies, there may be differences among the studies in terms of which specific activities they focus on. Table 1 lists a few core activities that are often included, to varying degrees, in such trials. However, when examining program content across trials, it becomes evident that although it would be interesting to examine studies according to dimensions of instructional features or activities, it is not straightforward to separate studies into different types of instruction.

**Table 1:** Description of instructional activities commonly used in intervention programs

Examples of activities commonly used in intervention programs
Activities that involve book reading (methodological approaches to this are often described as dialogic reading or interactive reading) Activities that use focus words as a gateway for discussions on selected topics (may be related to texts for older children) Activities that build narrative competence (either indirectly during storybook reading or directly by providing activities that activate work related to story structure) Activities that encourage active listening skills (listening to stories and answering questions after reading) Activities that encourage utilizing language (creating stories, explanations of/discussions on word meanings) Activities that involve direct vocabulary instruction: Direct instruction of words preselected for instruction Emphasizing words that occur in books or stories that are read for the children Encouraging discussions on word meanings Working with word consciousness (e.g., working with word parts and how they contribute to the overall meaning of a word) and grammatical understanding (e.g., reflecting on and manipulating the order of words in sentences)

#### 3.3.1 Instructor

In many of the intervention programs, particularly for preschool children, the teacher plays an important role in facilitating discussions during the intervention period. By providing definitions and examples, asking open‐ended questions, asking for clarifications, and engaging the children in active talk, children are encouraged to utilize active listening skills and express themselves. As evident from many intervention programs, participating teachers are instructed in strategies for facilitating high‐level discussions prior to the start of the intervention.

#### 3.3.2 Settings

The type of intervention programs that are included in this review provide children with language instruction sessions that are conducted in educational settings. This implies that the instruction programs can be considered as supplemental instruction, as the control group follows regular practice in preschools or school settings. However, studies may vary according to intensity (e.g., hours or days per weeks) and length of instruction (e.g., number of weeks).

### 3.4 How the intervention might work

#### 3.4.1 Theoretical background

At least three important theoretical perspectives set the stage for this review and are important in the discussion about how the intervention might work: The development of linguistic comprehension; the relationship between linguistic comprehension and reading comprehension; and the mechanism of why linguistic comprehension instruction should lead to transfer effects on outcomes of generalized language and reading comprehension skills.

##### The development of linguistic comprehension

The first perspective to be addressed is that linguistic comprehension appears to develop with a high degree of interdependence. Several cross‐sectional and longitudinal studies using observed variables have indicated that expressive and receptive vocabulary, grammar and syntax, and verbal memory are related skills that reflect a common factor (Colledge, et al., 2002; Johnson et al., 1999; MacDonald & Christiansen, 2002; Pickering & Garrod, 2013). This hypothesis has gained more conclusive support in large‐scale longitudinal studies that employ latent variables that correct for measurement errors: Bornstein et al. (2014) found a unitary core language construct from early childhood to adolescence. In addition, Klem et al. (2015) found a unidimensional latent language factor (defined by sentence repetition, vocabulary knowledge, and grammatical skills) in a longitudinal study of children aged 4–6 years. Further, recent studies that have included listening comprehension tests have also made arguments for a single language construct, in which different language assessment tools share a common variance. Justice et al. (2017) examined the development of language constructs in preschool through third‐grade children and reported that the latent variables “oral language” (indicated by receptive and expressive vocabulary and syntax) and “listening comprehension” (indicated by tests assessing the ability to comprehend narrative and expository passages as well as inferential skills) appeared to assess the same underlying construct. Similarly, a study by Lervåg et al. (2017) found that a latent language factor defined by vocabulary, grammar, verbal working memory, and inference skills was a clear predictor of the variation in “listening comprehension” measured by oral comprehension tests (explaining 95% of the variation in listening comprehension). Overall, these findings suggest that different language outcomes share a lot of common variance and that language skills, across domains (e.g., vocabulary and grammar) and modalities (expressive and receptive), are supportive of each other in development. A second important issue is the robust longitudinal stability within the linguistic comprehension domain. A stable rank order of children's vocabulary knowledge is preserved during both preschool and later school years (Melby‐Lervåg and Hulme, 2012; Storch & Whitehurst, 2002). The studies by Bornstein et al. (2014) and Klem et al. (2015) also indicate that the unitary core construct is highly stable over time. All these studies suggest that although all children's linguistic comprehension skills improve over time, the rank order between children is more or less preserved. This implies that altering children's language levels relative to other children is a complex and challenging endeavor. Nonetheless, as Bornstein et al. (2014) note, stability does not imply that it is impossible to change language skills through intervention. Thus, this review sheds light on important theoretical issues related to the nature of language learning, such as to what extent we—despite the high stability of linguistic comprehension and reading comprehension—can alter these skills and whether skills transfer from specific tasks integrated in the intervention to more generalized tasks in standardized tests.

##### The relationship between linguistic comprehension and reading comprehension

The second theoretical issue involves the relationship between our primary outcomes of linguistic comprehension and reading comprehension. How could improvement in linguistic comprehension transfer to reading comprehension? A close relationship between linguistic comprehension skills and the development of reading comprehension has been demonstrated in several longitudinal studies (Foorman et al. 2015; Lervåg et al., 2017; Torppa et al., 2016). Linguistic comprehension is a well‐known precursor to reading comprehension success, and it develops long before formal reading instruction begins (Hjetland et al., 2018; Snow, Burns, & Griffin, 1998). These studies align with the Simple View of Reading, which is a well‐established theoretical model of reading comprehension (Gough & Tunmer, 1986). This model presents reading comprehension as the product of decoding and linguistic comprehension skills and is formalized as the equation “Decoding × Linguistic comprehension = Reading Comprehension.” In this model, linguistic comprehension is an important underpinning in the development of reading comprehension beyond word‐level reading (Gough & Tunmer, 1986). While decoding is an important predictor of reading skills in the early reading phase, linguistic comprehension is understood as an essential predictor for the further development of reading comprehension (Hoover & Gough, 1990; Muter et al., 2004; Storch & Whitehurst, 2002). Studies on both second‐language learners and monolingual children with language delays have shown that the challenges they experience related to the understanding of texts are not characterized by a lack of decoding skills (Bowyer‐Crane et al., 2008; Spencer, Quinn, & Wagner, 2014). This indicates the importance of fostering linguistic comprehension skills to ensure proficient reading comprehension development. However, notably, at an older age (when linguistic comprehension explains the majority of variation in reading comprehension), reading comprehension has also proven to be a highly stable construct (Lervåg & Aukrust, 2010).

##### The mechanism of why linguistic comprehension instruction would lead to transfer effects on the outcomes of generalized language and reading comprehension skills

Theories on the nature of how and to what extent we can transfer what we learn are an important aspect of this review (see Bransford & Schwartz, 1999; Carraher & Schliemann, 2002). In this regard, two issues are at play: (a) the transfer of effects from criterion measures that contain the specific words that are used in the intervention to standardized tests of linguistic comprehension, and (b) the transfer of effects on linguistic comprehension to reading comprehension. Numerous studies indicate that children can easily be taught the meaning of novel words with which they are presented in an intervention (Elleman, Lindo, Morphy, & Compton, 2009). This phenomenon is often referred to as “near transfer.” However, in an intervention program, a child is typically presented with 3–6 novel words per week (Elleman et al., 2009). This amount is hardly sufficient to close the gap with children who have superior linguistic comprehension or the gap that exists between first‐ and second‐language learners because the comparison children also continuously develop their language skills. For example, among studies that provided direct vocabulary instruction that was either embedded in story book reading or as a separate component, it is important to note that there have been no intervention studies that have taught over 150 words or that have lasted over 104 hours (at least up until 2009; Elleman et al., 2009). Thus, for the studies that do show positive effects on generalized measures (e.g., Bowyer‐Crane et al., 2008; Clarke, Snowling, Truelove, & Hulme, 2010; Fricke, Bowyer‐Crane, Haley, Hulme & Snowling, 2013), it is not likely that instructing specific definitions of words is the causal factor that underpins this improvement. It is most likely that there are other factors in the instruction that led to the gains on standardized measures. Language interventions must teach children skills that are transferrable so that they can use them for general language development. These strategies can then be used when they encounter new words and unfamiliar sentences and not merely for the specific words taught in the intervention. As Taatgen (2013) stated, “Transfer in education is not necessarily based on content and semantics but also on the underlying structure of skills” (p. 469). Thus, to achieve long‐reaching transfer in language interventions (i.e., transfer beyond the specific words on which children are trained on more global language skills), an intervention must also focus on strategies that can be used in general language learning.

### 3.5 Why it is important to do the review?

Intervention programs have been designed to improve children's language skills. We know from earlier trials and prior reviews that children learn words that they have been directly instructed in. However, evidence on which educational intervention programs can produce effects on generalized language and reading comprehension tests that are not targeted for the specific intervention (distal effects) has been unclear. Several of the previous meta‐analyses are also now outdated, and recent studies are not included. The incorporation of these new studies makes our review substantially different from earlier reviews. Below, we provide the rationale for this systematic review and highlight some core differences from this review in contrast to prior reviews.

#### 3.5.1 Type of outcome measures

Prior meta‐analyses showed that effect sizes on generalized measures of linguistic comprehension and reading comprehension are typically much smaller and less impressive than effect sizes on proximal measures (see e.g., Elleman et al., 2009; Marulis & Neuman, 2010). While measures that refer to generalized language include test items that have not been explicitly trained in an intervention, proximal tests are designed by researchers to reveal the effects of targeted instruction. Thus, custom measures provide information on whether children have learned something that has been explicitly covered in an intervention (e.g., directly trained words in an instruction program). However, the ultimate goal of language‐based interventions is to enable children to accelerate their further growth in linguistic comprehension and reading comprehension skills. If we are to narrow the gap between children with small and large vocabularies, we must focus on providing children with the skills that are necessary to continuously develop knowledge of new words. Thus, unraveling the important factors that contribute to this generalization of knowledge becomes essential.

In the analyses of a synthesized effect, several meta‐analyses on linguistic comprehension instruction have combined outcomes that target the knowledge of instructed words along with generalized outcomes of vocabulary (e.g., standardized tests; Marulis & Neuman, 2010; 2013; Mol, Bus, & de Jong, 2009; Swanson et al., 2011). This procedure makes it difficult to interpret the results because they represent a mix of outcomes designed to assess instructed words and tests of words that are not targeted in the intervention program. A test of taught vocabulary is likely to produce a much larger effect size in an experimental study than a test of general vocabulary skills. Slavin and Madden (2011) described taught vocabulary tests as inherent to treatment. In cases of vocabulary instruction, these outcomes check for an understanding of instructed words that only the treatment group is exposed to. To provide some examples, the overall combined effect size of vocabulary instruction in preschool children in Marulis and Neuman (2010, 2013) was 0.88 and 0.87, respectively, which is almost one standard deviation of vocabulary measures. Mol et al. (2009) reported an overall combined effect size of 0.62 for expressive vocabulary and 0.45 for receptive vocabulary, and Swanson et al. (2011) reported 1.02 for combined vocabulary outcomes. In contrast, Elleman et al. (2009) displayed a more moderate effect size of 0.29 when the effect was synthesized on purely generalized vocabulary outcomes (outcomes of taught vocabulary were excluded from the analyses). These findings suggest that the use of different practices of including or excluding treatment outcomes inherent in the analyses is likely to explain (at least partially) differential results among prior reviews.

#### 3.5.2 Types of intervention programs

Several meta‐analyses on the topic have exclusively examined the value of shared book reading (e.g., Blok, 1999; Bus, van Ĳzendorn, and Pellegrini, 1995; Mol, Bus, de Jong, & Smeets, 2008), whereas others have included several types of vocabulary interventions in addition to print‐based training (Elleman et al., 2009; Marulis & Neuman 2010). Similar to Elleman et al. (2009) and Marulis and Neuman (2010), this review includes training studies that focus on both shared book reading and other types of vocabulary instruction. This review also includes studies that contain a broad view of oral language instruction (e.g., instruction that focuses on listening comprehension, narrative skills, and morphology/grammatical skills (e.g., Fricke, Bowyer‐Crane, Haley, Hulme, & Snowling, 2013). Our review also differs from meta‐analyses that have focused on interventions that address reading comprehension strategy instruction (Davis, 2010) and that address decoding and fluency (Edmonds et al., 2009; Scammacca, Roberts, Vaughn and Stuebing, 2015).

#### 3.5.3 Participants

This review expands the current literature by incorporating training studies from both preschool‐ and school‐aged children. The studies included could be those that were conducted in preschool and later educational settings up to the end of secondary school. Notably, the U.S. National Early Literacy Panel (2008) studied shared‐reading interventions in children aged 0–5, and no studies examined the impact of intervention on reading as an outcome variable. Similarly, Marulis and Neuman (2010) targeted only the very early years of vocabulary development (birth through age 6) and did not include measures of reading comprehension. Elleman et al. (2009) examined the impact of vocabulary instruction on reading comprehension in school‐aged children, where the majority of the studies included instruction conducted in Grades 3–5. In addition, this review also deviates from the review by Elleman et al. (2009) in that it does not exclude samples with a high proportion of second‐language learners.

#### 3.5.4 Settings

An additional reason for this review is the need for more knowledge of the effect of linguistic comprehension instruction conducted in educational settings. Bus et al. (1995) and Mol et al. (2008) studied book reading in parent–child settings and excluded interventions implemented in educational settings. Blok (1999) and Elleman et al. (2009) included only instruction studies in educational settings, whereas Marulis and Neuman (2010) included training studies implemented in both home and educational settings. Our aim is to focus on language instruction conducted exclusively in educational settings, because these studies have the most relevance for educational policy and practice. Thus, interventions implemented by parents or in the child's home environment are not included in this review.

#### 3.5.5 Design

A large number of previous meta‐analyses included studies without an appropriate control group, for example, within‐subject designs. This review included information from RCTs and QEs with a control group and measures of baseline differences. The current review also examined measures of follow‐up effects because the practical value of such interventions depends on the extent to which intervention effects are lasting. In order to draw some comparisons to earlier reviews, the reviews of school‐aged children by Elleman et al. (2009) and Stahl and Fairbanks (1986) are well‐known studies that examined the effect of vocabulary instruction on reading comprehension outcomes. Stahl and Fairbanks (1986) reported a mean effect size of *d* = .30 for standardized measures of reading comprehension, which is a promising finding that implies transfer effects from vocabulary instruction to generalized tests that do not include the instructed words. However, as Elleman et al. (2009) indicated, Stahl and Fairbanks (1986) included studies with designs without control groups. In addition, Stahl and Fairbanks (1986) did not weight the data by sample size in their analysis, which resulted in the equal contribution of effects from all studies regardless of sample size. In contrast, Elleman et al.'s (2009) review presented an effect size of 0.10 for generalized reading comprehension tests. This indicates a less powerful finding of transfer effects to reading comprehension outcomes as compared to that found by Stahl and Fairbanks (1986). However, the applicability of this finding in Elleman et al. (2009) is limited by the fact that the majority of studies included in the analysis were based on studies between the years 1963 and 1982, approximately 30–50 years from today. Lastly, this review deviates from prior reviews (Elleman et al., 2009) in that it did not exclude studies where problems related to treatment fidelity were reported by the authors.

## 4 OBJECTIVES

### 4.1 The problem, condition, or issue

This systematic review examined the effects of linguistic comprehension intervention programs and their effects on measures of generalized linguistic comprehension and reading comprehension.

The review aimed to answer the following main questions:

1. *Do linguistic comprehension intervention programs improve children's linguistic comprehension skills measured by generalized language outcomes?*
2. *Do linguistic comprehension intervention programs improve children's reading comprehension skills measured by generalized reading comprehension outcomes?*
3. *Which factors are associated with the impact of linguistic comprehension instruction on linguistic comprehension and reading comprehension outcomes?*
4. *What is the long‐term effect of linguistic comprehension intervention programs?*
5. *What is the separate effect on differential language constructs (e.g. vocabulary outcomes; grammar; narrative skills)?*

## 5 METHODS

### 5.1 Criteria for considering studies for this review

#### 5.1.1 Research design

Only control‐group designs were eligible. Both RCTs and QEs were included. RCTs that included the use of a controlled postexperimental design (without pretest assessment) were included. The QEs that were included conducted both pre‐ and posttest assessment. In addition, QEs with nonrandom assignment provided evidence that there were no baseline differences that were judged to be of substantial importance. This implies that a QE had to be matched or it had ensured that there was no inequivalence in demographic variables, such as socioeconomic indices for areas, parents’ income level, age, gender, or ethnicity. Studies using regression discontinuity design were not included.

#### 5.1.2 Years of publication

Studies from January 1986 until 2018 were eligible for inclusion. The reason we focused on the last 30 years is that it is important that the educational settings in which the studies are conducted are comparable over time.

#### 5.1.3 Intervention characteristics

- Studies that were included had to include an instructional method that targeted linguistic comprehension skills. Further, vocabulary training studies and studies incorporating vocabulary instruction within a more extensive approach to linguistic comprehension instruction (e.g., activities fostering grammatical knowledge, listening comprehension, and narrative skills) were eligible.
- Studies were included if they reported small additional elements of phonological awareness or letter knowledge instruction in their programs. However, the main focus had to be on the meaning‐based (semantic) aspect of language.
- Studies that only trained in phonological skills or grammatical skills (e.g., morphology or syntax) with the aim of improving phonological awareness and decoding skills were not considered to be eligible.
- Studies that focused on the improvement of linguistic comprehension by targeting broader cognitive skills, such as working memory or auditory processing, were not eligible and were considered beyond the scope of this review (e.g., Melby‐Lervåg & Hulme, 2012; Strong, Torgerson, Torgerson, & Hulme, 2011).

#### 5.1.4 Control conditions

Control conditions represented no treatment, waiting list treatment, or treatment as usual. Studies that compared different types of instructional methods for linguistic comprehension instruction were not eligible (e.g., two different approaches to teaching vocabulary). If the control condition included a type of instruction that targeted some other language constructs (e.g., phonological awareness) that related to the outcome measure of linguistic comprehension, the study was not included. Similarly, studies that included comparison groups that conducted alternative treatment that could impact reading comprehension outcomes were not included in the review (e.g., comprehension strategy instruction).

#### 5.1.5 Types of participants

Samples included participants from preschool and educational settings up to the end of secondary school. Groups of unselected typically achieving children, second‐language learners, children with language delay/problems, or children at risk for language and reading problems for other reasons (e.g., low socioeconomic backgrounds) were included in the study. Further, children with a special diagnosis such as autism or other physical, mental, or sensory disabilities were not eligible to be included in the review.

#### 5.1.6 Types of outcome measures

Studies that were included in the review were those that reported an intervention effect on at least one of the following two primary outcome variables:

##### Primary outcomes

- *Linguistic comprehension*: Reported outcomes had to be measured using tests that included items that had not been explicitly trained for in an intervention (e.g., standardized tests—tests created for research purposes that include items that were not instructed for in the intervention). Further, eligible outcomes could include both expressive and receptive tests of linguistic comprehension (e.g., tests of listening comprehension, grammar, vocabulary skills, narrative skills, and language composite tests that tap several language dimensions).
- *Reading comprehension*: Reported outcomes had to be measured using tests that included test items that had not been explicitly trained in an intervention (e.g., standardized tests—tests created for research purposes that include items not trained in the intervention).

##### Secondary outcomes

- Outcomes of follow‐up effects (delayed posttest) were coded if they were reported in the studies. The effect was then estimated from baseline to the follow‐up measurement point. We did not set any criteria for duration of follow‐up measurement timepoint to be extracted. In general, we expected that few trials would report the follow‐up measurement of effect.

#### 5.1.7 Types of settings

Studies that included training provided in educational settings were eligible for inclusion. To be included, an intervention had to be conducted in a day‐care center, preschool, kindergarten, or school setting. The intervention could be delivered by a teacher, assistant, or project staff (researcher or assistants associated with the research team). It could be provided within a classroom setting, in groups outside the classroom, or individually. Interventions implemented by parents or in children's home environments were not included. Further, interventions implemented in an educational setting plus home condition/homework were not eligible. This exclusion of parent–child studies was primarily because we wanted to be able to provide information on how intervention programs should be constructed in educational settings. There are several additional rationales underlying this choice of settings. As a group, parents do not have the pedagogical education or experiences that are likely to be present for providing the instruction in educational settings. Another important factor is that differences among parents in terms of educational background are likely to influence how the children will benefit, and numerous studies on parent–child book reading (which is the most common method of home‐based linguistic comprehension instruction) do not control for what actually happens in the control and experimental groups (Mol et al., 2008).

##### Search methods for identification of studies

The literature search was conducted in collaboration with information retrieval specialists at the Library of Human and Social Sciences, University of Oslo. Details of the search strategy and hits in bibliographical databases are provided in Supporting Information 1.

#### 5.1.8 Electronic searches

The electronic search was conducted in March 2016, and was limited to include references back to January 1986. In October 2018, an update identical electronic search was conducted to include studies between March 2016 and October 2018.

Studies were identified by searching the following electronic databases:

- Eric (Ovid)
- Psych INFO (Ovid)
- ISI Web of Science
- Proquest Digital Dissertations
- Linguistics and Language Behavior Abstracts (LLBA)
- Scopus Science Direct
- Bielefeld Academic Search Engine (BASE)
- Open Grey

The search was adapted to each database. Details on the search strategy for each database are provided in Supporting Information 1. The search limits included publications reported in English and dating back no more than 30 years from the original search.

#### 5.1.9 Searching other resources

##### Google scholar and relevant web‐pages

The search for literature also included specific search and screening of relevant hits on Google scholar (see Supporting Information 1). In addition, searches for gray literature included searches in relevant web‐pages, leading to authors in the field who were contacted for unpublished or in‐press manuscripts.

##### Hand search

Searches were conducted in prior meta‐analyses (Blok, 1999; Elleman et al., 2009; Fukkink & de Glopper, 1998; Goodwin & Ahn, 2010; Lonigan, Shanahan, & Cunningham, 2008; Marulis & Neuman, 2010, 2013; Mol et al., 2009; Pesco & Gagné, 2017; Stahl & Fairbanks, 1986) and in the following key journals: *Journal of Research in Reading*, *Journal of Research on Educational Effectiveness*, *Journal of Child Psychology, and Psychiatry*.

### 5.2 Data collection and analysis

#### 5.2.1 Selection of studies

**Figure 1:**
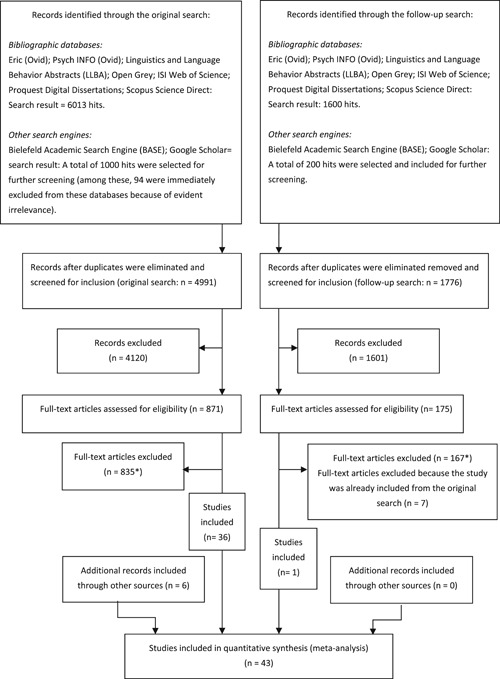
Flow diagram of the search and inclusion of references

The flow diagram in Figure 1 provides details of the search and selection of studies. For this review, the original searches were conducted in 2016, followed by a follow‐up search in 2018.

##### Original search

Our electronic search resulted in 6013 hits in the following databases: Eric (Ovid); Psych INFO (Ovid); Linguistics and Language Behavior Abstracts (LLBA); Open Grey; ISI Web of Science; Proquest Digital Dissertations; Scopus Science Direct. References were imported to EndNote for a duplication test. Since the Bielefeld Academic Search Engine (BASE) and Google Scholar does not allow for advanced search strings and result in a large number of hits, we chose to include the first 500 hits in each database for further screening. Of these, 94 references were immediately excluded because of clear irrelevance before importing references to the EndNote library (this was done because the importation of references from these databases to the EndNote library was less straightforward than for the other databases).

After duplication tests in EndNote, the remaining 4991 references were imported into the Distiller SR software program (Distiller SR, 2017) to screen eligible studies.

Studies were screened for inclusion or exclusion at the following three levels:

##### 5.2.1.1 Level one: Screening of abstract

At level one, 550 abstracts (random selection) were double screened by two of the authors. Inter‐rater agreement was assessed using Cohen's *κ, κ* = 0.73. References in conflict and questions regarding eligibility criteria were discussed before the remaining studies were divided and screened by the first and second authors.

##### 5.2.1.2 Level two: Screening of full texts

A large number of references did not report sufficient information in the abstract to make acceptable judgments about whether or not they met our criteria for inclusion. Overall, 871 of the 4,991 references in the distiller program were screened by examining the full‐text document of the study. A random sample of 20% of the remaining references was double screened at this level. Cohen's *κ*  was *κ* = 0.88. Questions related to eligibility criteria were discussed in the research group before the remaining references were screened for eligibility. The discussion on this mainly related to the following aspects of program content that were not predefined in the protocol: (a) hits that related to teacher professional programs—these type of programs were excluded, (b) hits that related to programs reporting reading comprehension outcomes but only contained a very small amount of linguistic comprehension instruction—we decided that studies had to be defined as programs with the main focus on linguistic comprehension skills (at least 50% amount of instruction). That implies that programs could be excluded if they were defined as mainly meta‐cognitive instruction or other strategy instruction.

##### 5.2.1.3 Level three: Additional screening of abstracts

In retrospect, because the original agreement at level one was low, we randomly selected 10% of all excluded references to examine whether it was likely that studies could have been missed out in level one. The concern regarding missed studies at level one is also related to the large number of studies that underwent single screening. In order to examine if studies could have been missed at this stage, the first and second authors double‐screened all the selected 10% of excluded references to judge if any of them should be included instead of being excluded. However, none of the references were changed from being excluded to included. Hence, this indicates that it was not likely that there were any missed references at the level one stage.

##### Follow‐up search

A follow‐up search was conducted in 2018. Our electronic search resulted in 1,600 hits in the following databases: Eric (Ovid); Psych INFO (Ovid); Linguistics and Language Behavior Abstracts (LLBA); Open Grey = 0 hits; ISI Web of Science; Proquest Digital Dissertations; Scopus Science Direct. For the BASE and Google Scholar, we included 100 hits in each database for further screening. In addition, we searched for studies in the following key journals: *Journal of Research in Reading*, *Journal of Research on Educational Effectiveness*, *Journal of Child Psychology, and Psychiatry*. The follow‐up search detected several studies that was already included but resulted in one additional study being included.

#### 5.2.2 Data extraction and management

##### Calculation of effect sizes

We calculated effect sizes by dividing the differences in gain between the pre and posttests in the treatment and control groups by the pooled standard deviation for each group at pretest; this method of effect size calculation for pretest–posttest designs is recommended by Morris (2008). Effect sizes for follow‐up tests were calculated in an analogous manner (pretest to follow‐up). When the effect size is positive, the group receiving linguistic comprehension instruction made greater pretest–posttest gains than the control group. In a few cases, the reported *F*‐statistic data were used to calculate the effect size (if mean differences and standard deviations in the treatment and control groups were not reported). When only posttest assessment was available (only in RCTs), we calculated the effect sizes by dividing the difference in means by the pooled standard deviation at posttest (or follow‐up). In one case, we only had posttest scores available with information to extract an effect size using the standard deviation of the control group.

We adjusted the effect size for small samples using Hedges’ *g* (Hedges & Olkin, 1985). *d* can be converted to Hedges’ g by using the correction factor J, corresponding to the following formula: J = 1 − (3/(4 *df* − 1; Borenstein, Hedges, Higgins, & Rothstein, 2009). The overall effect sizes were estimated by calculating a weighted average of individual effect sizes using a random effects model using 95% confidence intervals. Since the intervention studies were likely to differ in terms of sample characteristics, instructional features, and implementation of the programs, we selected a random effect model for estimating the effect. By choosing a random effect model for the analyses, the weighted average takes into account that the studies are associated with variations. In contrast to the fixed effect model where it is assumed that one true effect size underlies all the studies, the random effects model enables the effect sizes to vary from study to study (Borenstein et al., 2009). All effect sizes were double‐coded, and the first and second authors coded the information from the studies. Questions related to the coding of information were discussed in the research group.

#### 5.2.3 Measures of treatment effect

A few studies with multiple independent comparisons reported data from treatment and control groups from which data were not extracted for our study (e.g., parent‐based interventions). As noted in the protocol, only intervention and control groups that met the eligibility criteria were included.

##### Coding of effect size outcomes

In a large number of cases, the studies in this review included more than one linguistic comprehension outcome. All types of outcomes were coded and categorized into subtest categories of linguistic comprehension. Table 2 presents the types of linguistic comprehension outcomes that were coded, test descriptions and all corresponding tests used in the studies.

**Table 2:** Categories of outcomes, test descriptions and corresponding tests

Outcome categories	Test descriptions	[Corresponding tests used in the studies]
**Linguistic comprehension outcomes**		
Receptive vocabulary	Tests that require responses like pointing to pictures.	[PPVT; P‐CTOPP (subtest) The 40‐item receptive vocabulary; BPVS; CELF (subtest) Basic Concepts]
Expressive vocabulary	Tests that require expressive responses to name or explain the meaning of words (e.g., definition tests).	[WISC vocabulary (subtest); WASI vocabulary (subtest); WPPSI vocabulary (subtest); EOWPVT; BAS Naming Vocabulary; APT information; CELF (subtests) Expressive vocabulary, Word definitions, associations; WJ (subtest) Picture Vocabulary; ITPA (subtests) Verbal Expression, Verbal Fluency]
Reading vocabulary	Tests that examine the knowledge of words using an individual paper and pencil method (e.g., association tests—linking a word to one or more synonyms).	[GRADE word meaning; Gates‐MacGinitie vocabulary; Standford Vocabulary; SAT‐10; TORC‐3 Social studies vocabulary; ITBS vocabulary]
Composite vocabulary	Tests where the scores are represented by both a receptive and an expressive response.	CELF (subtest) Word Classes combined expressive and receptive score; TOLD Semantic composite (subtests: picture vocabulary, relational vocabulary, oral vocabulary)
Grammar	Test of grammatical knowledge (e.g., morphological awareness and grammatical understanding of sentences).	[APT grammar; CELF (subtest) Formulating Sentences, Sentence Structure; Researcher‐Created Morphology test; TROG; ITPA Grammatic Closure]
Narrative and listening comprehension	Tests defined as narrative tests (e.g., retelling tasks) or listening comprehension (typically, tests where the child listens to a story and is asked to respond to questions afterward).	[TNL (subtests) narrative comprehension, oral narration; Bus Story Information, MLU; YARC Listening Comprehension; OWLS Listening Comprehension; Researcher created story retelling tasks described as narrative comprehension, analyzed by MLU, number of words used, number of different words used, comprehension monitoring; GRADE listening comprehension; Researcher created tasks described as listening comprehension measures]
Language composite	Tests that were reported as composite language skills tests, with scores corresponding to several of the constructs mentioned above.	[BAS Verbal Comprehension; Children's Spontaneous Language, Complex utterances, rate of noun use, number of different words; upper bound index; CELF Core Language Composite Score (Sentence structure; word structure and expressive vocabulary); Preschool Language Assessment Instrument I and II—General Literal Language, Preschool Language Assessment Instrument III and IV—General Inferential Language]
**Reading comprehension outcomes**		
Reading comprehension		[GRADE passage comprehension; Gates‐MacGinitie Reading Comprehension; Standford Comprehension; WIAT reading comprehension; NARA reading comprehension; YARC‐Reading comprehension; New Group Reading Test [NGRT Passage comprehension score; ITBS reading comprehension/passage reading]

*Note*: Effect sizes extracted from researcher‐created measures are not developed based upon directly instructed words.

Abbreviations: APT, Action Picture Test; BAS, British Ability Scales; BPVS, British Picture Vocabulary Scale; CELF, Clinical Evaluation of Language Fundamentals; EOWPVT, Expressive One‐Word Picture; GRADE, The Group Reading and Diagnostic Evaluation; Vocabulary Test; ITBS, Iowa Test of Basic Skills; ITPA, Illinois Test of Psycholinguistic Abilities; MLU, Mean length utterance; OWLS, Oral and Written Language Scales; PPVT, Peabody Picture Vocabulary Test; TNL, Test of narrative language; TORC, Test of reading comprehension; TROG, Test of Reception of Grammar; WIAT, Wechsler Individual Achievement Test; WISC, Wechsler Intelligence Scale for Children; WPPSI, Wechsler Preschool and Primary Scale of Intelligence; WJ, Woodcock‐Johnson: YARC, York Assessment of Reading for Comprehension.

##### Managing data extraction

To analyze data, effect sizes and study information were entered into the “Comprehensive Meta‐Analysis” program by Borenstein et al. (2014). Data on risk of bias were entered into the software program Review Manager (Review Manager [RevMan], 2014) for summarizing and presentation of the results.

#### 5.2.4 Assessment of risk of bias in included studies

Two coders independently assessed the risk of bias for each study. Each category (selection bias, performance bias, detection bias, attrition bias, and reporting bias) was classified as high risk, unclear risk, or low risk, in accordance with Higgins, Altman, and Sterne (2011). Discrepancies were discussed and decided by consensus. Online supplement 3 provides details on the judgments and classification of the risk of bias categories.

#### 5.2.5 Unit of analysis issues

##### Multiple independent subgroup reporting

Dependency in effect sizes is a case when researchers report data from multiple independent subgroups. A few studies in this review reported separate data from different sites or separate analyses of children from different grades with separate control groups. For instance, two studies reported data from two subgroups that differed in grades and ages (Apthorp et al., 2012; Block & Mangieri, 2006), and two studies reported results from subgroups who differed in terms of implementation quality (Block & Mangieri, 2006; Lonigan & Whitehurst, 1998). According to Borenstein et al. (2009), when there are independent subgroups within a study, each subgroup contributes information that is independent and provides unique information. Therefore, we decided to treat each subgroup as though it was a separate study. This means that the independent subgroups were the unit of analysis.

##### Reporting of multiple outcomes

Several of the studies reported multiple effect sizes for outcomes of interest. There was no justification for including some outcomes and excluding others. At the same time, it was important to categorize outcomes into different language constructs in order to conduct sensitivity analyses. Therefore, we chose to code all language outcomes given for each study. In the overall effect analysis of linguistic comprehension instruction, a mean overall effect of reported outcomes is calculated so that each study only contributes one effect size.

In the sensitivity analyses of separate effects on different language constructs, all tests that corresponded to the given analyzed construct (Table 2) are merged, and each study only contributes one effect size in each analysis.

##### Multiple group comparisons

There were cases in which studies reported several treatment groups compared to the same control group (Dockrell, Stuart, & King, 2010; Fricke et al., 2017; Johanson & Arthur, 2016; Lonigan, Purpura, Wilson, Walker, & Clancy‐Menchetti, 2013; Silverman, Crandell, & Carlis, 2013). In these cases, where multiple experimental groups in one study matched the eligible criteria for inclusion, we computed a mean effect size from these studies in order to avoid them being treated as separate effects in the analyses.

#### 5.2.6 Dealing with missing data

##### Contacting authors in the field for clarification

Authors were contacted in cases where:

- Outcome measures were not standardized tests and it was unclear whether the test included items designed to measure the specific effect of instructed words or not.
- Several of the included references were sufficiently similar (due to intervention description and authors) to assume that they could be describing the same study.
- It was necessary to obtain more information in order to calculate effect sizes.

#### 5.2.7 Assessment of heterogeneity

Homogeneity *Q*‐tests were used to test the homogeneity of effect sizes using random effects models. The null hypothesis is that all studies share a common effect size. When the *p*‐value, set at .05, leads to a rejection of the null hypothesis, it suggests that the studies do not share a common effect size (Borenstein et al., 2009). The *Q*‐statistic is highly dependent on sample size. Therefore, in addition to calculating *Q*, we report *τ* ^2^ to examine the magnitude of variation in effect sizes among studies (Hedges & Olkin, 1985). *τ* ^2^ is used to assign weights in the random effects model and, thus, the total variance for a study is the sum of the within‐study variance and the between‐studies variance. This method for estimating the variance among studies is known as the method of moments (Borenstein et al., 2009). In order to quantify the impact of heterogeneity and assess inconsistency, we used the *I²* index. *I* ^2^ is used to examine what portion of the total variance in the effect sizes is due to true variance between the studies (Cooper, 2017). This quantity describes the percentage of the total variation across studies that is due to heterogeneity rather than chance and is recommended as a measure of heterogeneity (Higgins, Thompson, Deeks & Altman, 2003).

#### 5.2.8 Data synthesis

Since the intervention studies were likely to differ in terms of sample characteristics, instructional features, and implementation of the programs, we employed a random effects model to estimate the effect in all analyses. By selecting a random effects model for the analyses, the weighted average takes the variation associated with the studies into account (Borenstein et al., 2009).

Primary outcomes:

*Linguistic comprehension*: We examined the effect of the included intervention programs by synthesizing generalized outcomes of linguistic comprehension. For this analysis, all types of linguistic comprehension outcomes were included and synthesized to an overall mean effect size (see Table 2).
*Reading comprehension*: We also synthesized the effect of the included intervention programs on generalized outcomes of reading comprehension.

Secondary outcomes:

*Follow‐up effects*: All follow‐up effects were coded and synthesized to examine whether the effect of instruction was maintained over time.

#### 5.2.9 Subgroup analysis and investigation of heterogeneity

In the investigation of heterogeneity, the studies were divided into subsets of categorical moderator variables. Analyses were run using a random effects model and a *Q*‐test was used to examine whether the effect sizes differed between subsets. The overlap between confidence intervals was used to examine the size of the difference among the subsets of studies. Because of the limited number of training studies that examine follow‐up effects, moderator analyses were solely conducted for immediate posttest effects of linguistic comprehension and reading comprehension outcomes.

The following a priori moderators were examined.

##### Participant characteristics

- In terms of age, several studies reported intervention programs that spanned over several grade levels. Therefore, for the subgroup analyses, studies were categorized into the following grade levels: Preschool (including day care centers and corresponding to ages up to 5; kindergarten to second grade (starting from age 5); and sixth to eight grade levels (approximately 10 years or older).

##### Program dosage

- Total number of sessions. This variable was not normally distributed, and the total number of sessions was coded and categorized into “less than 50 sessions,” from 50 sessions to 100 sessions,” and “from 100 sessions or more.”
- Total hours of instruction. This variable was not normally distributed, and the duration of the intervention program was coded into the total number of hours of instruction and categorized into either less than 30 hr of instruction or 30 hr or more of instruction.
- Total number of weeks. This variable was not normally distributed, and the length of programs was coded in total number of weeks. Studies that only reported a duration of one academic year were coded as 30 weeks. For the analyses, studies were categorized into either “less than 20 weeks” or “20 weeks or more” of instruction.

##### Methodological characteristics

- Design. The studies were coded either as RCTs or QEs. Studies that only reported partial randomization, re‐assignment or referred to a very limited numbers of participants or blocks in the randomization process were coded as quasi‐experimental. Based upon findings from prior reviews (Cheung & Slavin, 2016), we were interested in examining whether QEs showed larger effect sizes than RCTs.
- Instructor. Studies were coded into categories of whether the intervention program was led by school personnel (e.g., teachers, teacher assistants) or project staff (researchers or persons affiliated with the research team). Programs implemented by project staff were hypothesized to be related to larger effect sizes than programs implemented by school staff.
- Small group or classroom instruction. Intervention programs implemented in small groups with less than 10 children were coded as small groups. Larger groups of children (10 or more) were coded as classroom instruction. Effect sizes from small group instruction were hypothesized to show larger effect sizes than classroom instruction.
- Implementation quality. All studies were assessed and judged to fall into one of the following categories: “no apparent problems, possible problems, and clear problems,” which are categories used in the study by Wilson, Tanner‐Smith, Lipsey, Steinka‐Fry, & Morrison, 2011). Information from the authors about possible problems, monitoring of the intervention, and whether this might have influenced the result was considered when judgments were made. Because there is no clear division between these categories of implementation quality, we used a set of judgment rules to assess the studies, as displayed in Table 3. Studies with clear implementation problems were hypothesized to show a smaller effect than studies without implementation problems.
  **Table 3:** Judgment of implementation quality

Judgment	Judgment rules
No apparent problems	The study reports implementation examination and does not emphasize that any implementation problems might have influenced the result. *OR* The study does not report implementation examination but reports monitoring strategies of the implementation and does not report implementation problems that might have influenced the result. *OR* The study does not report implementation examination or monitoring, yet the program are implemented by researchers themselves. No report that implementation problems might have influenced the result.
Possible problems	The authors indicate possible problems with implementation of the program and report that this may have influenced the result.
Clear problems	The authors indicate clear problems with the implementation and clearly state that they expect this to have influenced the result of the trial.

#### 5.2.10 Sensitivity analyses

Sensitivity analyses were conducted to examine how the results would be affected by assumptions in the analyses. We examined the following type of analyses and corresponding questions:

(1) *Changing the unit of analysis approach*: What is the summary effect on linguistic comprehension and reading comprehension if independent comparisons within studies are not treated as the unit of analysis but instead combined to a mean composite effect size within each study (using studies as the unit of analysis)?
(2) *Multiple stratified analyses of risk of bias*: What is the summary effect on linguistic comprehension if studies of high or unclear risk of selection and attrition bias are removed, thereby leaving only the studies with low risk in the analyses?
(3) *Sensitivity analysis of posteriori moderator*: Is study location (studies from Europe vs. US/North America) related to the effect on linguistic comprehension?
(4) *Multiple stratified analyses of differential language outcomes*: What is the effect of instruction on differential language outcomes?

#### 5.2.11 Publication bias analyses

Publication bias occurs when a mean effect size is upwardly biased because only studies with large or significant effects are published (i.e., the file‐drawer problem with entire studies) or because authors only report data on variables that show effects (often referred to as *p*‐hacking, or the file‐drawer problem for parts of studies; see Simmons, Nelson, & Simonsohn, 2011; Simonsohn, Nelson, & Simmons, 2014). In order to statistically estimate how publication bias can impact results, funnel plots are often used in combination with a trim‐and‐fill analysis. However, this funnel plot/trim‐and‐fill method has several methodological weaknesses (Lau, Ioannidis, Terrin, Schmid & Olkin, 2006). Therefore, in order to analyze publication bias in this review, we selected a p‐curve analysis instead. *p*‐Curve analysis is a recently developed method that addresses the weaknesses in funnel plot/trim‐and‐fill analysis (Simonsohn et al., 2014). A *p*‐curve plots the distribution of statistically significant *p*‐values (*p* < .05) in published studies, and the shape of the *p*‐curve is a function only of the effect size and sample size. In the presence of true effects, one expects the distribution of published *p*‐values to be right‐skewed with a larger number of low *p‐*values (.01 s) than high *p‐*values (.04 s) In contrast, if a set of studies is affected by publication bias (because researchers discard entire studies or discard analyses or parts of studies), the *p*‐curve becomes left‐skewed or flat. Such a p‐curve is said to provide “no evidential value” (i.e., no support for an appreciable effect size). When interpreting findings from *p*‐curve analyses, it must be noted that there are several approaches to addressing publication bias and selective reporting bias (Ioannidis, Munafo, Fusar‐Poli, Nosek, & David, 2014; Sterne, Becker, & Egger, 2006), and the *p*‐curve method is a relatively new method for assessing *p‐*hacking. The *p*‐curve method has also been criticized for not addressing a substantial amount of heterogeneity, not providing a confidence interval, and not testing for publication bias (van Aert, Wicherts & van Assen, 2016).

### 5.3 Deviations from the protocol

Some main differences between the protocol and the review must be noted:

- Inclusion of studies: With regard to research designs, no specific criteria was mentioned within RCTs and QE studies. In this review, we did not include regression discontinuity design. We also chose not to include quasi‐experimental studies where baseline differences between the groups were judged to be of importance but not accounted for.
- Inclusion of studies: With regard to inclusion of control groups, this review only includes treatment as usual/waiting list/or irrelevant play‐based control group settings. We did not include control groups that received alternative treatment corresponding to language activities—for example, any other type of book reading or instruction in phonological awareness or letter knowledge instruction.
- Examination of moderator variables related to program characteristics: Not all moderators described in the protocol are present in the review. We intended to examine whether there were differences among studies in terms of how the intervention program provided definitional and contextual instruction and whether they maintained a low or high levels of discussion during the training session. The included studies showed little variance on these aspects. Variables related specifically to the monitoring of program instruction as well as education and experience among teachers implementing the intervention were also difficult to obtain from all studies and were not included.
- Examination of moderator variables related to sample characteristics: Not all moderators related to sample characteristics described in the protocol were included in the review (SES and language status). This is described in the results section, when discussing the sample characteristics of the included studies.

## 6 RESULTS

### 6.1 Description of studies

#### 6.1.1 Results of the search

The search strategy resulted in the inclusion of 43 studies. Table 4 presents an overview of all studies with key information on program, design, language of instruction, and the instructor of each intervention. Table 5 displays a summary of key characteristics of the studies that were included.

**Table 4:** Overview of included studies, type of publication, instruction program, language of instruction, and setting

Study	Design^a^	Publication	Instruction program	Language of instruction	Setting	Instructor leading the program
Apthorp (2006)	RCT	Published article	Supplemental vocabulary program	English	3rd grade, Title 1 schools (United States)	Teacher
Apthorp et al. (2012)	RCT	Published article	Supplemental vocabulary program	English	Kindergarten, 1st grade, 3rd grade, 4th grade (United States)	Teacher
Block and Mangieri (2006)	RCT	Published article	Powerful vocabulary for reading success	English	3rd grade, 4th grade, 5th grade, 6the grade (United States)	Teacher
Brinchmann et al. (2015)	QE	Published article	Word knowledge instruction	Norwegian	3rd, 4th grade (Norway)	Teacher
			Working with texts, meanings, and morphology			
Cable (2007)	QE	Unpublished dissertation	Narrative instruction	English	2nd grade (United States)	Researcher
Clarke et al. (2010)	RCT	Published article	Oral language program	English	Year 4 classrooms (United Kingdom)	Teaching assistants
Coyne et al. (2010)	QE	Published article	Direct and extended vocabulary instruction	English	Kindergarten (United States)	Teacher/grad students
Crain‐Thoreson and Dale (1999)	RCT	Published article	Shared book reading	English	Preschool (United States)	Teacher/librarian/aide/nurse
Dockrell et al. (2010)	QE	Published article	Intervention group 1: Talking time	English	Preschool (United Kingdom)	Teacher/assistant
			Intervention group 2: Story reading			
Farver, Lonigan, and Eppe (2009)	RCT	Published article	Literacy Express Preschool Curriculum English only	English	Head Start Preschools (United States)	Graduate research assistant
Fricke et al. (2013)	RCT	Published article	Oral language intervention	English	Nursery schools (United Kingdom)	Teaching assistant
Fricke et al. (2017)	RCT	Published article	Oral language intervention	English	Nursery and reception (United Kingdom)	Teaching assistant
Gonzalez et al. (2010)	RCT	Published article	The Words of Oral Reading and Language Development (WORLD)	English	Preschool (Head Start) (United States)	Teacher/assistant
Hagen, Melby‐Lervåg, and Lervåg (2017)	RCT	Published article	Language Comprehension Intervention	Norwegian	Preschool (Norway)	Teacher
Haley, Hulme, Bowyer‐Crane, Snowling, and Fricke (2017)	RCT	Published article	Oral Language Intervention	English	Preschool (United Kingdom)	Teacher
Johanson and Arthur (2016)	QE	Published article	Intervention group 1: Let's know deep Intervention group 2: Let's know broad	English	Pre‐K (United States)	Teacher
Justice, Mashburn, Pence, and Wiggins (2008)	RCT	Published article	The Language‐focused Curriculum	English	Preschool (United States)	Teacher
Justice et al. (2010)	QE	Published article	Read it again! Language and literacy instruction	English	Preschool (United States)	Teacher
Kelley, Goldstein, Spencer, and Sherman 2015)	QE	Published article	Storybook intervention with vocabulary and question‐answering lessons (pre‐recorded storybooks)	English	Pre‐K (United States)	Teacher
Lawrence et al. (2015)	RCT	Published article	Word Generation	English	Middle school classrooms (United States)	Teacher
Lawrence, Francis, Paré‐Blagoev, and Snow (2017)	RCT	Published article	Word Generation	English	Middle school classrooms (United States)	Teacher
Lesaux, Kieffer, Faller, and Kelley (2010)	QE	Published article	Academic Language Instruction for All Students	English	6th grade (United States)	Teacher
Lesaux, Kieffer, Kelley, and Harris (2014)	RCT	Published article	Academic Language Instruction for All Students	English	6th grade (United States)	Teacher
Lonigan and Whitehurst (1998)	QE	Published article	Shared Reading Intervention (Dialogic Reading)	English	Child Care Centers (United States)	Teacher
Lonigan et al. (2013)	RCT	Published article	Group 1: Dialogic Reading plus PA Training Group 2: Dialogic Reading plus LK Training Group 3: Dialogic Reading plus PA/LK Combined	English	Preschool (United States)	Project staff
Lonigan, Anthony, Bloomfield, Dyer, and Samwel (1999)	RCT	Published article	Dialogic (shared) reading	English	Preschool (United States)	Graduate volunteers
Murphy et al. (2016)	QE	Published article	The Vocabulary Enrichment Program (adapted version)	English	Secondary School (Ireland)	Teacher
Neuman, Newman, and Dwyer (2011)	RCT	Published article	World of Words Word Knowledge and Conceptual Development (multimedia approach)	English	Preschool (Head Start) (United States)	Teacher
Nielsen and Friesen (2012)	QE	Published article	Small group intervention on vocabulary and narrative development	English	Kindergarten (United States)	Doctoral student
Phillips et al. (2016)	RCT	Published article	Literate language intervention	English	Title 1 Pre‐kindergarten (United States)	Teacher
Pollard‐Durodola et al. (2011)	RCT	Published article	Words of Oral Reading and Language Development intervention (WORLD)	English	Preschool (Head Start) (United States)	Teacher
Proctor et al. (2011)	QE	Published article	Improving Comprehension Online: Deep Vocabulary Instruction (internet‐delivered)	English Spanish	5th grade (United States)	Teacher
Rogde, Melby‐Lervåg, and Lervåg (2016)	RCT	Published article	General language instruction	Norwegian	Kindergarten (Norway)	Teacher
Schaefer et al., unpublished	RCT	Unpublished manuscript	Oral language intervention	English	Primary School (United Kingdom)	Teaching assistant
Silverman et al. (2013)	RCT	Published article	Intervention group 1: Read aloud plus Intervention group 2: Read aloud	English	Head Start Preschool (United States)	Teacher
Simmons et al. (2010)	RCT	Published article	A Multiple‐Strategy Approach to Content‐Area Vocabulary Instruction	English	4th grade (United States)	Teacher
Spencer et al. (2014)	QE	Published article	Narrative intervention	English	Head Start Preschool (United States)	Teacher
Styles and Bradshaw (2015)	RCT	Published article	The Vocabulary Enrichment Program and the Narrative Intervention Program.	English	Year 7 (United Kingdom)	Teacher assistant
Vadasy, Sanders, and Logan Herrera (2015)	RCT	Published article	Rich vocabulary instruction	English	4th and 5th grade classrooms (United States)	Teacher
Valdez‐Menchaca and Whitehurst (1992)	RCT	Published article	Dialogic reading	Spanish	Day Care Center (Mexico)	Graduate student
van Kleeck, Vander Woude and Hammett (2006)	RCT	Published article	Scripted book‐sharing discussions	English	Preschool (Head Start) (United States)	Project staff
Wasik and Bond (2001)	QE	Published article	Interactive book reading and book reading extension activities.	English	Title 1 Preschool (United States)	Teacher
Whitehurst et al. (1994)	RCT	Published article	Picture book reading	English	Day Care Centers (United States)	Teacher or aid

Abbreviations: QE, quasi‐experiment; RCT, randomized controlled trial.

^a^ The design type (RCT, QE) was coded by the review authors.

**Table 5:** Characteristics of included studies

Publication and design characteristics			Program and implementation characteristics		
Publication characteristics	Mean	*SD*	Program characteristics	*k*	%
*Publication years 1992–2017*	2010	6.3	*Instructor*		
			Project staff	7	16
*Publication type*	*k*	%	School staff	36	84
Journal article (published)	40	94			
Report (published)	1	2	*Sample language status*	*k*	%
Dissertation	1	2	Can't tell	10	23
Unpublished article	1	2	Monolingual sample^b^	10	23
			Linguistically diverse sample	20	47
*Study location*	*k*	%	Second language learners only	3	7
Europe	11	26			
US/North America	32	74	*Instruction setting*	*k*	%
			Classroom instruction > 10	20	47
Design characteristics^a^			Instruction in smaller groups < 10	23	53
*Assignment*	*k*	%			
Randomized controlled trial	28	65	*Program dosage*	mean	sd
Quasi‐experiment	15	35	Hours of instruction	68	52
			Number of sessions	69	52
*Control group activities*			Weeks of implementation	18	10
Treatment as usual/waiting list control	41	95			
Described as other play activities	2	5	Implementation quality^c^	*k*	%
			No apparent problem	34	69
			Possible problems	9	19
			Clear problems	6	12

^a^ Coded by the review authors.

^b^ One study reported that second‐language learners were omitted from the sample before analyzing the results.

^c^ Independent subgroups within studies is the unit of report.

All 41 studies are published articles or published reports. With regard to the low number of gray literatures, it must be noted that there were a substantial number of hits related to unpublished dissertations that were screened for eligibility. However, they were typically excluded because of deviations from our inclusion criteria related to design issues or lack of generalized outcome measures. We also detected one ongoing study Joffe et al., in preparation.

##### Program characteristics

The search identified studies of linguistic comprehension instruction conducted with day‐care, preschool, kindergarten, and school‐aged children. The studies conducted with preschool children, before reading onset, typically involved elements of interactive book reading and/or direct instruction of linguistic comprehension skills (vocabulary, grammar, and/or narrative skills). A few of the studies conducted with preschool‐aged children included certain additional elements of early literacy instruction, such as phonological awareness and letter‐sound knowledge. In studies conducted with school‐aged children, the linguistic comprehension instruction typically involved vocabulary instruction within the context of reading experiences or/and discussions.

##### Sample characteristics

With regard to sample characteristics, we intended to examine whether there was a difference in effect among studies on how participants were selected for participation (like screening for language difficulties), low or high SES backgrounds, and language status, in order to conduct a separate analysis of second‐language learner samples. Regarding the SES background, multiple measures, differential and imprecise reporting, or failure to report SES made this information difficult to categorize (although it may be partially represented in other moderator variables that are included in the review, such as study location). Further, with regard to language status, it was unclear (no report) in approximately one‐fourth of the studies whether or not the sample included second‐language learners. In other studies, even though it was reported that second‐language learners were included, separate analyses for this group of students was not reported. At least two studies (Schaefer et al., unpublished; Spencer et al., 2014) reported that there were no differences in effects for participants with second‐language status and, therefore, did not report separate analyses. On account of this, the analyses in this review is based on a synthesized effect across sample characteristics.

##### Design

A total of 28 RCTs and 15 QE are included in the review. Some studies reported random allocation, continued to re‐allocate participants after randomization, or used a very limited number of clusters or individuals in their randomization process. Therefore, studies could be coded as a QE study even though they employ a randomization technique.

##### Implementation quality report in the included studies

A total of 6 independent subgroups reported that it was likely that poor implementation could have impacted the results, 9 independent subgroups were also judged to represent studies with possible problems, and 34 independent subgroups reported no apparent problems.

#### 6.1.2 Excluded studies

Detailed descriptions and references to excluded studies are provided in Supporting Information 2.

### 6.2 Risk of bias in included studies

**Figure 2:**
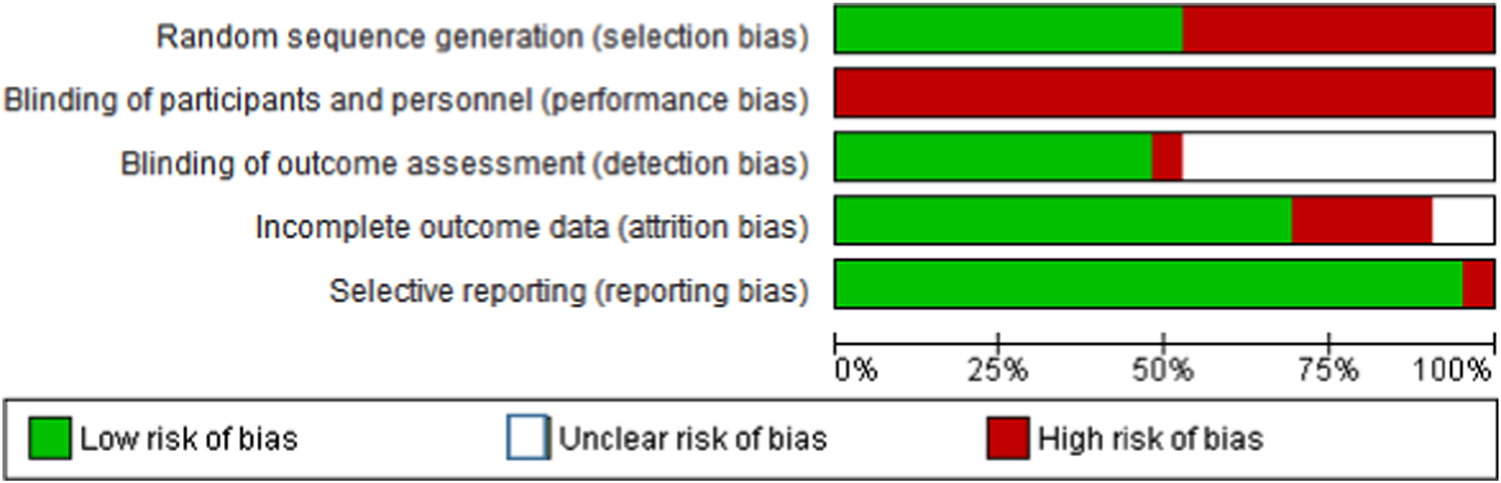
Risk of bias graph: Review authors’ judgments regarding each risk‐of‐bias item presented as percentages across all included studies

The risk of bias in the included studies was assessed by evaluating selection bias, performance bias, detection bias, attrition bias, and reporting bias. See Figure 2 for a summary of the risk of bias assessment, and Supporting Information 3 for a more detailed description of the risk of bias judgments. The following section summarizes the results from the assessments of risk of bias for each bias category. The section ends with information on how the quality of evidence is incorporated in the results.

#### 6.2.1 Selection bias

Differences due to selection and allocation were examined in all studies. If the study did not involve random allocation, selection bias was rated as high risk. For cluster RCTs, the number of schools and classrooms were taken into consideration for the judgment category. Even though several studies were labeled as RCTs or randomization was highlighted in describing the study design, studies could be rated as high‐risk on selection bias due to a small *N*, reporting of unequal groups, or a small number of classrooms/schools. Approximately half of the studies (23) were judged as having a low risk of selection bias and the other half (20) as a high risk of selection bias.

#### 6.2.2 Performance bias

None of the studies included in this review were conducted with blinded personnel or participants after enrollment into the study. All studies (43) represented a high risk for performance bias, thereby implying that there is a risk that the knowledge of which intervention treatment was received could affect the outcome.

#### 6.2.3 Detection bias

Approximately half (21) of the studies reported blinding of the outcome assessment. Only two studies reported non‐blinded assessment. Further, approximately half (20) of the studies did not report whether or not the assessments were blinded and were, thus, categorized as being unclear.

#### 6.2.4 Attrition bias

Most studies reported attrition rates. The reason for attrition was typically due to children shifting from schools. When making judgments about attrition bias, we examined the reasons for attrition. We also examined whether there were discrepancies between the treatment and the control groups due to attrition rates. The majority of studies (30) were judged as being at low risk of attrition bias. The remainder were judged as being at high risk of attrition bias (9), and a few were judged as having an unclear risk (4) status of attrition bias.

#### 6.2.5 Reporting bias

A majority of the studies were classified as “low risk of bias,” as no indication of reporting bias was detected. One study was classified as high risk because data for one of the outcomes of interest were not reported for the total sample due to non‐significant findings in certain portions of the sample.

#### 6.2.6 Quality of the included studies: Issues of analysis and presentation

No threshold was defined to exclude studies related to specific criteria for high risk of bias. This implies that all studies were included without any stratification incorporated into the main analyses. Even though stratification could have presented a result with less bias, the rationale underlying not defining a threshold was that including only studies with a low risk of bias in a domain could produce a result that is imprecise if there are few high‐quality studies (Higgins et al., 2011). On account of unclear information in numerous studies related to risk of bias, we did not wish to create an overall quality index based on all obtained information on quality. Calculating a summary score may result in an inconsistent product, as the assessment of bias is criticized for focusing on the quality of reporting rather than the design and conduct of the study (Moher et al., 1995).

Thus, the risk of bias assessment was not incorporated in the overall mean analyses of effect on linguistic comprehension and reading comprehension outcomes. However, we conducted sensitivity analyses to examine the effect of instruction when only studies with low risk of selection bias and attrition bias were remaining in the analyses.

Notably, an issue that is not included in the risk of bias assessment is measurement error. However, this is clearly important, since measurement error is likely to attenuate effect sizes (Cole & Preacher, 2014). Unfortunately, very few of the studies reported coefficients of internal consistency and only a handful of studies used methods to correct for measurement error. Thus, it was not possible to use this as a moderator. However, this is clearly an important issue that has not been given sufficient attention in previous studies.

### 6.3 Synthesis of results: Primary outcomes

#### 6.3.1 The immediate effects of instruction on linguistic comprehension and reading comprehension outcomes

The effect of linguistic comprehension training was examined by analyzing the overall effect on linguistic comprehension outcomes and reading comprehension outcomes. Effect sizes and heterogeneity measures for the two outcomes are summarized in Table 6.

**Table 6:** Immediate effect of linguistic comprehension instruction on linguistic comprehension (overall) and reading comprehension (overall)

Outcome	*k*	*N* treatment	*N* control	*g* [95% CI]	*p*	Heterogeneity
Linguistic comp. (posttest)	48	13,567	12,146	0.16 [0.10, 0.22]	.0001	*Q* = 150.89; *df* = 47; *p* = .0001; *T* ^2^ = 0.02; *I* ^2^ = 68.85
Reading comp. (posttest)	16	9,758	8,045	0.05 [−0.01,0.12]	.13	*Q* = 37.38; *df* = 15; *p* = 0.001; *T* ^2^ = 0.007; *I* ^2^ = 59.87

**Figure 3:**
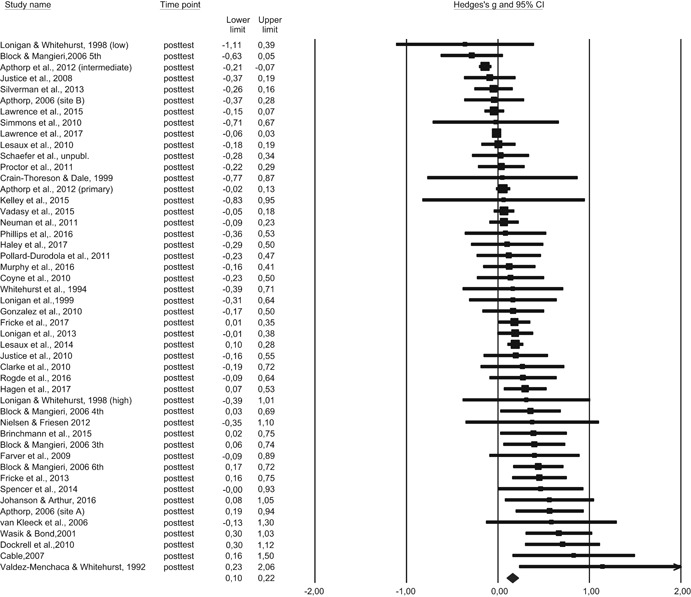
Forest plot of the effect of linguistic comprehension instruction on linguistic comprehension outcomes posttest (overall)

The overall analysis of the effect on linguistic comprehension outcomes synthesized 48 effect sizes, comparing treatment and control groups. The mean effect size was small in favor of the treatment groups (*g* = 0.16), with evidence of heterogeneity in the effect sizes. A forest plot of the analysis is presented in Figure 3.

**Figure 4:**
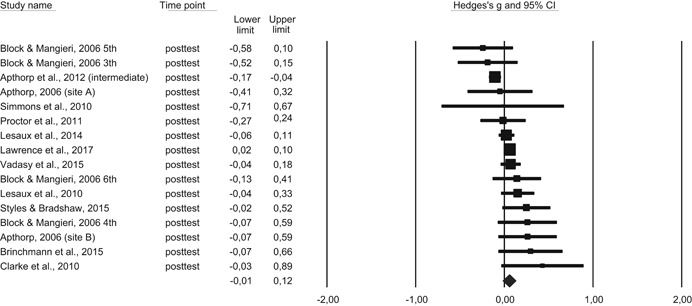
Forest plot of the effect of linguistic comprehension instruction on reading comprehension outcomes posttest (overall)

The overall analysis of the effect on reading comprehension outcomes synthesized 16 effect sizes comparing the treatment and control groups. The mean effect size was close to zero (*g* = 0.05), with evidence of heterogeneity in the effect sizes. A forest plot of the analysis is illustrated in Figure 4.

### 6.4 Subgroup analyses and investigation of heterogeneity

Subgroup analyses were conducted for the overall linguistic comprehension outcome and reading comprehension outcome.

#### 6.4.1 Moderators of effect on linguistic comprehension (overall)

Table 7 presents the results of the moderator analyses. There were no significant differences in effect sizes between studies in terms of grade or on variables related to dosage of instruction. With regard to variables related to methodological characteristics, quasi‐experimental designs yielded significantly higher effect sizes than randomized controlled designs. Effect sizes were also found to be related to implementation quality, in which studies with no apparent implementation problems displayed a statistically significant larger mean effect size than studies with possible or clear problems. In addition, as shown in Table 7, the mean effect size was higher for project‐led programs than for programs led by school staff, and higher for programs implementing the program in small groups than instruction provided in classroom settings.

**Table 7:** Moderators of effect on linguistic comprehension (overall): Number of effect sizes, effect size, 95% confidence interval (CI), heterogeneity statistics, and *p*‐values for the test of difference test among categories

Moderator variable	Number of effect sizes (*k*)	Effect size (*g*)	95% CI	Heterogeneity (*I* ^2^)	Test of difference (*Q*‐test)
**Grade**					
Preschool (under 5 years of age)	26	0.21**	[0.12, 0.30]	33.74	
Kindergarten to fifth grade	15	0.16*	[0,03, 0,30]	77.37	
Sixth to eighth grade	7	0.07	[−0.02, 0.18]	74.84	0.14
**Total hours of instruction** ^a^					
Less than 30 hr of instruction	21	0.21**	[0.11, 0.31]	10.10	
30 hr of instruction or more	26	0.14	[0.07, 0.21]	78.25**	0.26
**Total number of sessions** ^a^					
Less than 50 sessions	20	0.23**	[0.12, 0.34]	14.90	
50–100 sessions	13	0.17**	[0.02, 0.20]	51.89	
100 sessions or more	13	0.11**	[0.02, 0.20]	84.42	0.36
**Total number of weeks**					
Less than 20 weeks	29	0.16**	[0.08, 0.25]	39.99**	
20 weeks or more	19	0.15**	[0.07, 0.23]	81.11**	0.84
**Design**					
QE	16	0.31	[0.15, 0.46]	54.10**	
RCT	32	0.12	[0.05, 0.18]	69.74**	0.02**
**Implementation quality**					
No apparent problems	34	0.24**	[0.16, 0.32]	72.34**	
Possible problems	8	0.01	[−0.07, 0.10]	36.05	
Clear problems	6	−0.03	[−0.17, 0.11]	3.02	0.0001**
**Instructor**					
School staff	41	0.14**	[0.10, 0.20]	69.75**	
Project staff (researchers etc.)	7	0.37**	[0.15, 0.60]	25.58	0.05*
**Group size instruction**					
Classroom instruction (11 or more)	25	0.10**	[0.03, 0.17]	75.01**	
Groups (1–10)	23	0.25**	[0.17, 0.33]	3.20	0.004**

Abbreviations: QE, quasi‐experiment; RCT, randomized controlled trial.

^a^ Studies with multiple group comparisons and differential reports of dosage among multiple intervention groups have been excluded from the analysis.

* *p* < .05.

** *p* < .01.

#### 6.4.2 Moderators of effect on reading comprehension (overall)

We conducted moderator analyses of effect on the reading comprehension outcome despite an overall small effect size. Table 8 presents the results of the moderator analyses. Like the analyses of linguistic comprehension outcomes, grade level and program dosage (total number of hours, sessions, and weeks of instruction) were not found to be moderators of effect. With regard to methodological characteristics, all the studies with reading comprehension outcomes represented programs that had been led by school staff. Only 3 of the 16 studies had a quasi‐experimental design. These studies represented a larger mean effect size than RCTs, but the difference was not statistically significant. Implementation quality was not a moderator of effect. Among the studies, only 3 reported instruction in groups, thereby showing a statistically significant larger effect size than programs with instruction provided in classroom settings.

**Table 8:** Moderators of effect on reading comprehension (overall): Number of effect sizes, effect size, 95% confidence interval (CI), heterogeneity statistics, and p‐values for the test of difference test between categories

Moderator variable	Number of effect sizes (*k*)	Effect size (*g*)	95% CI	Heterogeneity (*I* ^2^)	Test of difference (*Q*‐test)
**Grade**					
Kindergarten to fifth grade	10	0.03	[−0.10, 0.17]	52.69*	
Sixth to eighth grade	6	0.06**	[0.03, 0.10]	0.00	0.66
**Total hours of instruction**					
Less than 30 hr of instruction	14	0.06	[−0.25, 0.22]	0.00	
30 hr of instruction or more	2	−0.02	[−0.01, 0.13]	65.10**	0.55
**Total number of sessions**					
Less than 50 sessions	4	0.12	[−0.02, 0.30]	0.00	
50–100 sessions	4	0.08	[−0.08, 0.25]	51.77	
100 sessions or more	8	0.02	[−0.07, 0.11]	71.40	0.45
**Total number of weeks**					
Less than 20 weeks	6	0.07	[−0.04, 0.17]	16.07	
20 weeks or more	10	0.06	[−0.03, 0.15]	70.32**	0.88
**Design**					
QE	3	0.12	[−0.02, 0,27]	4.66	
RCT	13	0.04	[−0.04, 0.12]	64.06**	0.33
**Implementation quality**					
No apparent problems	9	0.07	[−0.04, 0.17]	67.93**	
Possible problems	6	0.07**	[0.02, 0.11]	0.00	
Clear problems	1	−0.24	[−0.58, 0.10]	0.00	0.21
**Group size instruction**					
Classroom instruction (11 or more)	13	0.03	[−0.04, 0.10]	59.44**	
Groups (1–10)	3	0.30**	[0.10, 0.50]	0.00	0.01**

Abbreviations: QE, quasi‐experiment; RCT, randomized controlled trial.

* *p* < .05.

** *p* < .01.

### 6.5 Synthesis of results: Secondary outcomes

#### 6.5.1 The long‐term effects of instruction on linguistic comprehension and reading comprehension outcomes

The long‐term effect of linguistic comprehension instruction was examined by analyzing the overall effect on linguistic comprehension outcomes and reading comprehension outcomes. Effect sizes and heterogeneity statistics for the two outcomes are presented in Table 9.

**Table 9:** Long‐term effect of linguistic comprehension instruction on linguistic comprehension (overall) and reading comprehension (overall)

Outcome	*k*	*N* treatment	*N* control	*g* [95% CI]	*p*	Heterogeneity
Linguistic comp. (follow‐up)	8	737	722	0.23 [0.09, 0.36]	0.001	*Q* = 10.76; *df* = 7; *p* < .15; *T* ^2^ = 0.01; *I* ^2^ = 34.94
Reading comp. (follow‐up)	4	468	466	0.33 [0.00, 0.65]	0.05	*Q* = 14.85; *df* = 3; *p* = .002; *T* ^2^ = 0.084; *I* ^2^ = 79.79

**Figure 5:**
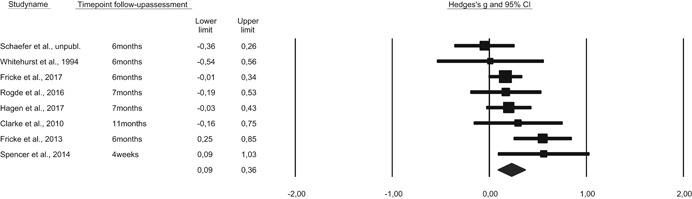
The effect of linguistic comprehension instruction on linguistic comprehension outcomes at follow‐up (overall)

The overall analysis of the long‐term effect on linguistic comprehension outcomes synthesized eight effect sizes, comparing the treatment and control groups. The mean effect size was small in favor of the treatment groups (*g* = 0.23), with evidence of heterogeneity in the effect sizes. A forest plot of the analysis, with timepoints for follow‐up assessment, is depicted in Figure 5.

**Figure 6:**
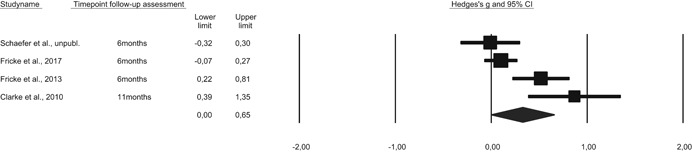
The effect of linguistic comprehension instruction on reading comprehension outcomes at follow‐up (overall)

The overall analysis of the long‐term effect on reading comprehension outcomes synthesized four effect sizes, comparing treatment and control groups. The mean effect size was small (*g* = 0.33), with evidence of heterogeneity in the effect sizes. A forest plot of the analysis is presented in Figure 6.

### 6.6 Sensitivity analyses

#### 6.6.1 Changing the unit of analysis approach

In the analysis of primary outcomes, independent subgroups within a study were the unit of analysis and they were treated as though they were a separate study. Therefore, we examined what the synthesized immediate effect would have been if we instead combined all reported independent comparisons within studies and used study‐level as the unit of analysis. Table 10 presents the results of the sensitivity analysis. There was no noteworthy difference in the summary overall effect on linguistic comprehension outcomes or reading comprehension outcomes when we changed the unit of analysis.

**Table 10:** The effect of instruction on linguistic comprehension and reading comprehension with study‐level as the unit of analysis: Number of effect sizes, effect size, 95% confidence interval (CI), and heterogeneity statistics

Changing the unit of analysis								
Outcome	*k*	*g*	95% CI	*p*	*Q*(*df*)	*p*	*I* ^2^	*τ* ^2^
Linguistic comprehension (overall)	42	0.15	[0.09, 0.21]	0.0001	110.53 (41)	.0001	62.91	0.01
Reading comprehension (overall)	12	0.06	[−0.01, 0.13]	0.12	29.31 (11)	.002	62.48	0.01

#### 6.6.2 Multiple stratified analyses of risk of bias

We examined what the summary immediate effect on linguistic comprehension (overall) would have been if studies of high or unclear risk of selection bias and attrition bias were excluded, thereby leaving only the studies with low risk in the analyses. Table 11 presents the result of the sensitivity analysis (independent subgroups within a study is the unit of analysis).

**Table 11:** The immediate effect of instruction on linguistic comprehension (overall) in studies with low risk of selection and attrition bias: number of effect sizes, effect size, 95% confidence interval (CI), and heterogeneity statistics

Effects on linguistic comprehension (posttest) stratified to low‐risk studies								
Risk status	*k*	*g*	95% CI	*p*	*Q*(*df*)	*p*	*I* ^2^	*τ* ^2^
Studies with low risk of selection bias	27	0.11	[0.04, 0.18]	0.0001	92.23(26)	.0001	71.81	0.04
Studies with low risk of attrition bias	31	0.19	[0.01, 0.28]	0.0001	94.92(30)	.0001	68.40	0.03

With regard to low risk of selection bias, the mean effect on linguistic comprehension would have been small (mean effect size *g* = 0.11) in favor of the treatment groups if only low risk studies of selection bias was included in the analysis (a decrease in mean effect size from the main analysis). The analysis summarizing studies with only a low risk of attrition bias shows that the effects would have increased (from *g* = 0.16 to *g* = 0.19) if only studies with low risk of attrition bias were included.

#### 6.6.3 Analysis of posteriori moderator: Study location

In the main analyses, we decided to synthesize the effect from programs both in the United States and Europe. Therefore, we summarized the effect of instruction on linguistic comprehension stratified to Europe versus United States/North America studies and examined if the effect varied between the study locations. Table 12 presents the result from the sensitivity analysis. Studies located in Europe displayed a statistically significant larger mean effect size than studies from the United States/North America.

**Table 12:** Study location as a moderator of effect on linguistic comprehension (overall): number of effect sizes, effect size, 95% confidence interval (CI), heterogeneity statistics, and p‐values for the test of difference among categories

Moderator variable	Number of effect sizes (*k*)	Effect size (*g*)	95% CI	Heterogeneity (*I* ^2^)	Test of difference (*Q*‐test)
*Study origin*					
Europe	10	0.26**	[0.15, 0.37]	20.43	
United States/North America	38	0.12**	[0.06, 0.19]	68.75*	0.04**

* *p* < .05.

** *p* < .01.

#### 6.6.4 Sensitivity analysis: Multiple stratified analyses of linguistic comprehension instruction on differential language outcomes

We examined the immediate (posttest) and follow‐up effect of linguistic comprehension instruction on multiple stratified analyses of differential categories within linguistic comprehension outcomes (for an overview of outcomes and test types, see Table 2). The results from the analyses are presented in Table 13 (see Supporting Information 5 for a forest plot of each analysis when more than four effect sizes are synthesized). When a study reported multiple indicators for the same construct, the means of the indicators were computed. The results showed that a synthesis of narrative and listening comprehension outcomes showed moderate immediate effect sizes at posttest (*g* = 0.42), represented by 13 studies. The effect of instruction, when synthesized across all vocabulary outcomes, showed a small immediate mean effect size (*g* = 0.13). Similarly, the effect of instruction on outcomes of grammatical understanding was small (*g* = 0.17).

**Table 13:** Separate effects on differential language outcomes of linguistic comprehension

	Effect size [95% CI]			Heterogeneity		
Outcome	(g) [95% CI]	*p*	*Q*	*p*	*I* ^2^	*r* ^2^
**Vocabulary, timepoint (no. of studies)**						
Vocabulary overall (composite all vocabulary outcomes, posttest) (*k* = 43)	0.13 [0.07, 0.19]	.0001	112.49	.0001	62.66	0.02
Vocabulary overall (composite all vocabulary outcomes, follow‐up) (*k* = 7)	0.17 [0.03, 0.31]	.02	9.05	.17	33.70	0.01
Reading vocabulary, posttest (*k* = 14)	0.05 [−0.03, 0.13]	.19	52.72	.0001	75.34	0.012
Expressive vocabulary, posttest (*k* = 23)	0.17 [0.10, 0.24]	.0001	24.10	.34	8.70	0.00
Expressive vocabulary, follow‐up (*k =* 7)	0.21 [0.07, 0.35]	.004	9.12	.17	34.20	0.01
Receptive vocabulary, posttest up (*k* = 19)	0.19 [0.09, 0.29]	.0001	25.99	.10	30.74	0.01
Receptive vocabulary, follow‐up up (*k* = 4)	0.07 [−0.06, 0,19]	.30	2.07	.56	0.00	0.00
Vocabulary composite, posttest (*k =* 2)	0.14 [−0.12, 0.40]	.30	0.002	.96	0.00	0.00
**Narrative and listening comprehension, timepoint (no. of studies)**						
Narrative and listening comprehension, posttest (*k* = 13)	0.42 [0.25, 0.58]	.0001	49.29	.0001	75.66	0.06
Narrative and listening comprehension, follow‐up (*k* = 6)	0.27 [0.15, 0.40]	.0001	6.35	.27	21.23	0.005
**Grammatical understanding, timepoint (no. of studies)**						
Grammar, posttest (*k* = 10)	0.17 [0.07, 0.28]	.001	15.22	.09	40.85	0.01
Grammar, follow‐up (*k* = 4)	0.25 [−0.12, 0.62]	.18	38.97	.0001	89.74	0.15
**Language composite, timepoint (no. of studies)**						
Language composite, posttest (*k* = 4)	0.20 [−0.25, 0.65]	.38	9.44	.024	68.20	0.13

### 6.7 Analysis of publication bias

In order to address publication bias, we performed a *p*‐curve analysis of published studies that reported statistically significant (*p* < .05, two‐tailed) effects of linguistic comprehension instruction on generalized measures. As outlined in the method section, when studies have evidential value, a *p*‐curve will have a larger number of low *p*‐values than high *p*‐values and will, thus, appear right‐skewed (Simonsohn et al., 2014). In the analysis that we conducted, a combination of half and full *p*‐curve analysis was used (Simonsohn, Simmons, & Nelson, 2015). Evidential value is indicated when the half *p*‐curve test is right‐skewed, with *p* < .05, or when both the half and full tests are right‐skewed with *p* < .1. If the studies have no evidential value, the *p*‐curve will be left‐skewed with a larger number of higher *p*‐values than lower *p*‐values. This may be interpreted as evidence for publication bias or *p*‐hacking (Simonsohn et al., 2014).

**Figure 7:**
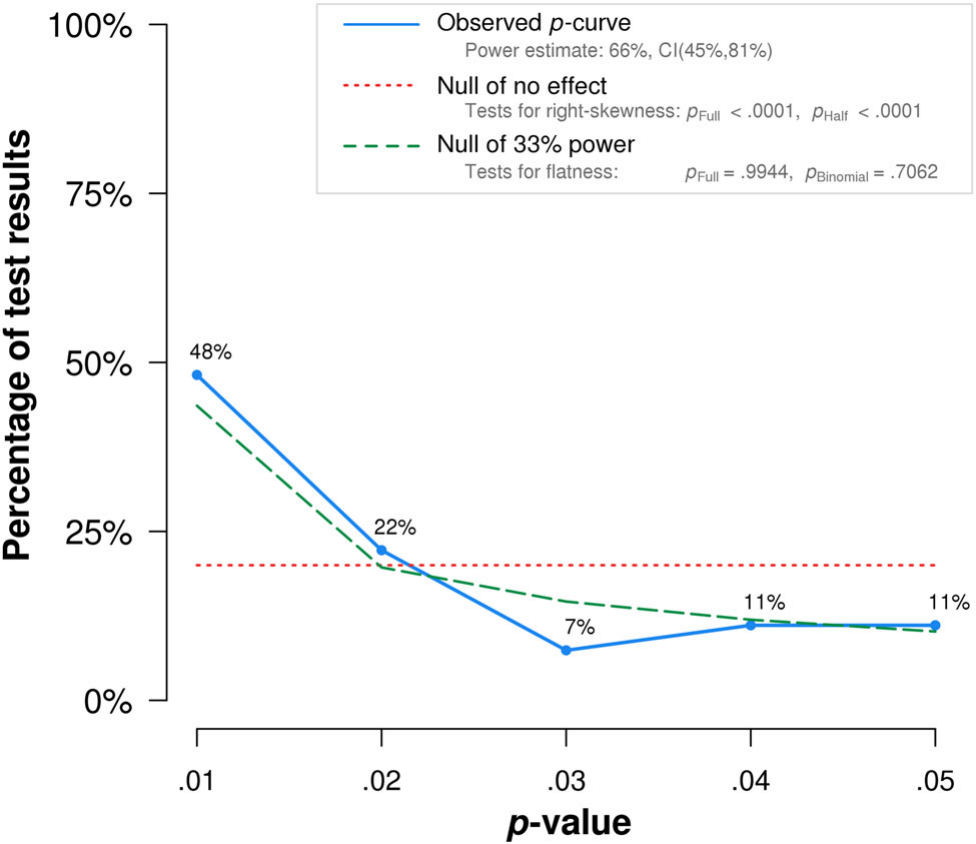
Results of the *p*‐curve analysis

Figure 7 presents the result from the *p*‐curve analysis. A total of 27 studies were included in the *p*‐curve analysis (see Supporting Information 4 for details on the studies the *p*‐curve is based on, as well as the excluded studies). Null of 33% power refers to the pattern that the figure would have if there was a true effect when studies had 33% power and no publication bias/*p*‐hacking. This overlaps greatly with the observed *p*‐curve and is a good sign that there is no *p*‐hacking/publication bias. As depicted in Figure 7, the *p*‐curve is right‐skewed, thereby providing no evidence of publication bias. Both the half (*z* = −6.01, *p* < .0001) and the full (z = −6.18, *p* < .0001) *p*‐curve tests suggest the presence of evidential value. Thus, this analysis suggests that the studies included have evidential value, with 20 out of 27 *p*‐values being below .025. Notably, it is fairly common for published studies in this field to use one‐tailed significance tests. This was the case for several of the studies included in this systematic review. These *p*‐values were transformed into two‐tailed values in the *p*‐curve analysis. One of the results (Lonigan et al., 2013) exceeded a value of .05 when we used a two‐tailed approach and was, therefore, excluded from the *p*‐curve. In this case, the next reported statistically significant result that met our criteria was included in the *p*‐curve analysis instead.

## 7 DISCUSSION

The present review identified 43 studies evaluating the effects of linguistic comprehension instruction on generalized linguistic and reading comprehension outcomes. The review aimed to answer the following questions: (a) Do linguistic comprehension intervention programs improve children's linguistic comprehension skills measured by generalized language outcomes? (b) Do linguistic comprehension intervention programs improve children's reading comprehension skills measured by generalized reading comprehension outcomes? (c) Which factors are associated with the impact of linguistic comprehension instruction on linguistic comprehension and reading comprehension outcomes? (d) What is the long‐term effect of linguistic comprehension intervention programs? (e) What is the separate effect of linguistic comprehension instruction on differential language constructs (type of outcome used)?

### 7.1 Summary of main results

#### 7.1.1 The effect of instruction on generalized outcomes of linguistic comprehension and reading comprehension

The two first objectives of this review addressed whether linguistic comprehension instruction can improve children's linguistic comprehension and reading comprehension outcomes measured by generalized tests that are not designed based on target words instructed in a program.

The results indicated that linguistic comprehension instruction can have a positive immediate effect on linguistic comprehension outcomes, although with a small summary (mean) effect size (*g =* 0.16). As expected, these findings show that when solely generalized outcomes are synthesized, the results display a more restrictive pattern of effect sizes than when outcomes directly targeted to instructed words are incorporated in the analyses (Marulis & Neuman, 2010, 2013; Mol et al., 2009; Swanson et al., 2011).

The immediate effect on generalized measures of reading comprehension was negligible (*g* = 0.05). Even though the relationship between linguistic comprehension and reading comprehension is well situated in the literature, the absence of clear effects on generalized outcomes for the type of trials included in this review is in line with findings from the review by Elleman et al. (2009).

#### 7.1.2 Relationship between effect size and study characteristics

Our third objective was to examine variables that could impact the effect of linguistic comprehension instruction on the primary outcomes. In order to achieve with this, we conducted subgroup analyses to examine how moderator variables were related to the effect of instruction. However, it is important to note that moderator analyses are not causal and can only indicate whether a type of study characteristic is associated with a higher or lower effect size (see Hedges & Pigott, 2004).

The overall analyses of treatment effect on linguistic comprehension and reading comprehension showed that instruction in groups tends to be more effective than instruction provided in whole classroom settings. It is reasonable to state that smaller instruction groups are associated with larger effects than large instruction groups; however, when examining other studies in terms of this aspect, the picture is not entirely clear (Elleman et al., 2009; Neuman & Kaefer, 2013; Vaughn et al., 2010).

Contrary to expectation, there was no difference in the treatment effect on linguistic comprehension or reading comprehension related to the dosage of the intervention programs. This might reflect the fact that the effects on the outcomes of interest were generally small. In addition, it is important to note that the coding of dosage (total number of hours, sessions, and weeks) is based on the intended pattern of instruction in the included studies. It is possible that actual implementation may have deviated from this prescription, thereby yielding imprecise results.

The mean effect size for programs instructed by project staff was larger than for programs implemented by school staff on the linguistic comprehension outcome. This difference was not statistically significant; however, it is not unlikely that researcher‐instructed programs are implemented with higher fidelity than programs implemented by school staff.

Further, tendencies across the linguistic comprehension outcomes showed that securing implementation quality is an influential factor for obtaining effects in such studies. Nevertheless, the categorization of the implementation quality of studies was not straightforward. As methods for ensuring and assessing treatment fidelity differed across studies, it is difficult to be precise about the extent to which fidelity problems are in fact present in the studies. In addition, one cannot exclude the possibility that there may be a tendency to explain a lack of effect as a problem of implementation, or that those studies with clear effects may under‐report fidelity problems in attributing positive findings to the instruction program.

Given the diversity among samples, we checked for whether study location was related to the treatment effect on linguistic comprehension. Our results indicated variability in effect sizes associated with the locations of where the studies were conducted. This may be a function of European participants’ higher SES and lower cultural diversity as compared to those of U.S. samples. Another possible explanation is the more extensive use of smaller intervention groups in European studies.

Finally, our findings correspond to the results of Cheung & Slavin's (2016) review of methodological impacts on a large sample of educational evaluations, where they report statistical significantly higher effect sizes in QEs than in randomized experiments. The summary (mean) effect size on linguistic comprehension in QEs was statistically higher than that for RCTs. With regard to the reading comprehension outcome, the majority of effect sizes were derived from RCTs.

#### 7.1.3 The long‐term effect of linguistic comprehension instruction on linguistic comprehension and reading comprehension outcomes

In terms of the fourth objective—whether linguistic comprehension instruction has any long‐term benefit—the review revealed that only a few of the included studies reported follow‐up assessments. A total of eight effect sizes on linguistic comprehension contributed to the analysis, thereby showing a small positive effect size (*g* = 0.23) in favor of the treatment groups. Most likely, only studies with a clear picture of intervention effects at posttest choose to undertake further assessments of the participants in their sample. This may explain the pattern of a larger summary effect at this timepoint than at the immediate assessment point.

Only four effect sizes were extracted to examine the long‐term benefit on reading comprehension, displaying a small to moderate effect size (*g* = 0.33). Among these, only the study by Clarke et al. (2010) reported reading comprehension as an immediate outcome, with positive findings. The other three studies (Fricke et al., 2013, 2017; Schaefer et al., unpublished) implemented their program with preschool children; thus, reading comprehension was not assessed before a delayed posttest. Except for the studies by Fricke et al. (2013, 2017) and Schaefer et al. (unpublished manuscript), no other studies on young children reported follow‐up reading comprehension assessments, and the studies show divergent findings. It must also be noted that these studies reported additional instruction related to phonological awareness and letter knowledge. This may have influenced the results of the reading comprehension outcomes, particularly since the children were in their early years of reading instruction when the assessment took place.

#### 7.1.4 The effect of instruction on differential language constructs

The last objective was to examine the separate effect of linguistic comprehension instruction on differential language constructs. Therefore, we conducted multiple stratified analyses that corresponded to differential language constructs and outcomes measures reported in the studies. When the effect of instruction was synthesized across all vocabulary outcomes, the results showed a small immediate effect size (*g* = 0.13) in favor of the treatment groups. In addition, we also conducted separate syntheses of effects related to types of vocabulary outcomes. We found that the instruction programs showed relatively small positive immediate effects on generalized measures of receptive and expressive vocabulary (*g =* 0.19 and *g =* 0.17, respectively), while few studies reported follow‐up data. Further, intervention effects measured by vocabulary reading tests (word meanings examined by a paper and pencil method) on older children showed no evidence of effect.

Similar to the findings on expressive and receptive vocabulary outcomes, the effect of instruction on outcomes of grammatical knowledge showed small positive immediate effects in favor of the treatment groups (*g =* 0.17). Only four studies reported follow‐up effects on outcomes of grammatical knowledge, yielding divergent results.

The results showed that a synthesis of narrative and listening comprehension outcomes showed a moderate immediate effect size at posttest (*g* = 0.42), represented by 13 studies.

Follow‐up effects showed that there was a maintained but decreased effect of instruction on narrative and listening comprehension at subsequent timepoints (*g =* 0.27). It must be noted that the studies included in the analysis of narrative and listening comprehension were represented by samples of young children in preschool and early grades.

In summary, a larger effect size was reported for outcomes of narrative and listening comprehension skills than for generalized outcomes of vocabulary and grammar.

It is possible that these types of generalized outcome measures are more closely interwoven with the instruction than the typically generalized outcomes of receptive and expressive vocabulary. Narrative activities such as book reading, working with story elements, and discussions on words and content were explicitly reported as important instructional elements in virtually all the studies on preschool children that were included in our review. In addition, narrative comprehension tests and listening comprehension tests represent assessments that integrate knowledge across different language domains, and the format of these assessments is generally different from that of standard vocabulary and grammar tests. While narrative and listening comprehension test scores could be based on information from retelling or answering questions after listening to stories, vocabulary and grammar tests typically require responses such as pointing to pictures representing words or actions or explaining the meaning of single words with increasing difficulty. Thus, it is possible that elements represented in narrative and listening comprehension assessment tools are more sensitive to detecting changes in children's language development due to the intervention programs included in this review as compared to standard vocabulary and grammar tests. It is also the case that certain narrative and listening comprehension tests included in a few studies were created by researchers, even though they were not directly targeted to specific content or words instructed. Nevertheless, these assessment tools might be closer in context to the targeted instruction than other standardized outcomes of vocabulary and grammar that are not created for the same purpose. According to Cheung & Slavin (2016), there are good reasons to believe that measures designed by researchers are more sensitive to treatment than independent standardized tests, even when the tests are not inherent to the treatment.

Lastly, it must be noted that these analyses of effect are divided on the basis of type of outcomes reported. Therefore, several studies are represented in multiple analyses, if they reported various language outcomes, while some of the included studies are only represented in one of the analyses.

### 7.2 Overall completeness and applicability of evidence

In terms of this review, it is important to mention that since studies were classified according to different characteristics, the analyses were based on fewer studies, which resulted in lower statistical power. This can lead to non‐significant results and failure to detect moderator effects. In addition, unclear reporting in studies included in this review made it difficult to code important moderators that were originally planned and assumed to explain variances in effect sizes. This is a form of missing data in a meta‐analysis (Pigott, 2012). For example, multiple measures, differential and imprecise reporting, or failure to report SES made it difficult to categorize information. While a few studies reported school location or neighborhood‐based indicators of SES, others reported the (approximate) percentage of participants that receive free lunches, and others reported the number of students below the poverty line. Because of the difficulty of categorizing such information, SES was not included in the analyses, although it may be partially represented in other moderator variables that were included in the review (e.g., study location). With regard to sample characteristics, the results were also generalized across samples of monolingual students (typically selected on the basis of weak language skills) and samples of second‐language learners. Because numerous studies did not report separate analyses for second‐language participants in their sample and there were instances of studies reporting that this was not conducted as there were no differences in results from the monolingual participants, we did not discriminate results on the basis of differential sample characteristics.

Moreover, there is a lack of information about the educational level and experience of those implementing the programs, which was reported only in a few studies. The level of instruction prior to program implementation was also difficult to ascertain, as this information was imprecise or excluded in several cases.

With regard to the instructional programs, the review was unable to establish whether different approaches to instruction might explain the variation in results across the included studies. The instructions in the included studies tended to employ many of the same instructional elements but differed in terms of their emphasis on different methods (e.g., interactive reading, vocabulary instruction, and grammar instruction). Categorizing intervention programs in terms of different forms of instruction would, in general, require more detailed information.

Lastly, it is important to note that this review summarized findings from studies that target linguistic comprehension instruction as the *main focus* of instruction (at least 50% of the instructional time). This raises an important issue due to the extensive range of interventions that target reading comprehension among school‐aged students. Multicomponent studies that incorporate a substantial amount of code‐related reading skills, strategy, and meta‐cognitive skills were not included in this review. In summary, a broader perspective on reading comprehension instruction must be included in discussions on potentially effective approaches to enhancing children's reading comprehension skills.

### 7.3 Quality of the evidence

The studies included in this review involved both RCTs and QE designs with a pretest–posttest design and a control group. When examining the risk of selection bias in the included studies, 23 studies were judged to be at low risk of selection bias. This left 20 studies, approximately half of the studies in this review, with a high risk of selection bias. Our sensitivity analysis displayed that if only RCT studies that were judged to be at low risk of selection bias had been included, the overall mean effect for linguistic comprehension would have been *g =* 0.11. Thus, it is possible that the mean effects could be upwardly biased.

Because it is not possible to blind participants and personnel in the experiments included in this review, all studies represented a high risk of performance bias. Thus, in every study, there was a risk for systematic differences among groups other than in the intervention programs that were conducted, which may lead to both underestimated and overestimated effect in trials.

Another type of bias that was present in this review is detection bias, as almost half of the studies did not report whether or not the assessment was blinded (which may be an indication of a lack of blinded assessment). If detection bias should occur, it is likely to result in an overestimation of effect sizes.

We also examined whether studies reported withdrawal or incomplete outcome data from participants at posttest assessment points, as this could indicate attrition bias, which is a type of selection bias that occurs after the treatment has taken place. A majority of the studies reported low attrition rates, but approximately one‐fourth of the studies reported issues related to attrition rates or did not provide sufficient information to make a judgment. In our sensitivity analysis, we excluded studies with high or unclear risk of attrition bias. This indicated that the mean summary effect on linguistic comprehension would have been *g* = 0.19 if only studies with low risk of attrition bias had been included.

The quality of the evidence also depends upon whether there is risk of contamination (spillover) effect in the trials included in the review. Even though a study employs randomization techniques, contamination can lead to reduction in treatment effects. Although the risk for this type of bias was not assessed, it is possible that it may still be present in some of the trials in this review.

Finally, the problem of missing studies is always a possible source of biased conclusions when conducting systematic reviews. The *p*‐curve analysis conducted for this study provided no evidence of publication bias.

In conclusion, there are possible biases associated with the studies included in this review that may have led to both overestimation and underestimation of the intervention effect in the trials. Therefore, the results of this review must be interpreted with caution.

### 7.4 Limitations and potential biases in the review process

A limitation of this review is that the search for literature was limited to publications reported in English, which may reduce precision of the results if relevant data are not included (Moher et al., 1996). In addition, our search detected few unpublished manuscripts eligible for inclusion. As previously noted, an extensive amount of unpublished manuscripts was collected and screened for eligibility but did not reach our inclusion criteria.

Another limitation of this review is that the effect of instruction is examined on observed variables. Because meta‐SEM requires a correlation matrix of the variables of interest from the primary studies, this was not an option for the analyses as too few studies reported this. Another option could have been to adjust for measurement errors by measuring the extent of random measurement and correcting for it (Schmidt & Hunter, 2014). In the given studies, estimates of reliability are typically reported using Cronbach's *α* to examine the internal consistency of test scores. However, because some studies did not report Cronbach's *α* values or simply referred to Cronbach's *α* values as reported in the test manual, it was not possible to correct for measurement errors in the analyses. Consequently, the estimated effect sizes are likely to be attenuated because of measurement error in the analyses.

Lastly, it is important to note that moderator analyses are not causal and can only say something about a relationship between the moderator variable and effect size. Thus, it is possible that any third variables not controlled for can explain a relationship or difference among study subsets.

### 7.5 Agreements and disagreements with other studies or reviews

Our review detected a small effect of instruction on generalized outcomes of linguistic comprehension and negligible effect on reading comprehension. The review by Elleman et al. (2009) reported a larger mean effect size for standardized vocabulary measures (*d =* .29). With regard to the low impact on outcomes of reading comprehension in the current study, the result is not surprising considering that the study by Elleman et al. (2009) detected an effect size of *d* = .10 on generalized reading comprehension outcomes.

## 8 AUTHORS’ CONCLUSIONS

### 8.1 Implications for practice and policy

The present findings showed that effect sizes for linguistic comprehension outcomes range from 0.10 to 0.22 for immediate effects and 0.09 to 0.36 for follow‐up effects. The results from the *p*‐curve analysis indicated that this is a true effect that is not limited by publication bias.

Providing meaningful descriptions of practical importance is not straightforward and must be done with caution (see Cooper, 2008, 2017). According to the What Works Clearinghouse (U.S. Department of Education, Institute of Educational Sciences, & National Center for Education Evaluation and Regional Assistance, 2014) and the Promising Practices Network (2014), an effect size of 0.25 standard deviations or larger can be considered “substantially important.” In contrast to this guideline, Hill, Bloom, Black, and Lipsey (2008) argued that there is a range of variations within educational interventions that will potentially have different implications for interpreting the practical magnitude of effect sizes. According to Hill et al. (2008), a claim that an effect size of 0.25 is required for educational significance has no general applicability.

Another way of interpreting the size of the effect is to calculate a derived *d* and transfer it into months of schooling. Lee, Finn, and Liu (2018) did this for reading based on U.S. data of national norms of academic growth. They suggested that the effect size for a reading program with *d* = 0.2 (i.e., 20% of one standard deviation) in kindergarten would be equivalent to 1 month of schooling (*d* = 0.1). The impact of this “small” effect increases as children grow up: The effect size of 0.2 would become worth 4 months (*d* = 0.4) in Grade 4, 1 year in Grade 8 (*d* = 1.0), and 3 years plus 4 months (*d* = 3.4) in Grade 12. Unfortunately, since language comprehension is not directly taught in school, we do not have similar figures for this outcome. They could have been calculated by dividing the treatment effect by the control group growth rate per school year. However, data for this were rarely reported in the included studies; therefore, this could not be estimated.

Further, as Lipsey & Wilson (1993) argued, it is important to underscore that small effects on educational treatments from meta‐analyses are not be dismissed as lacking practical or clinical significance. Our view is that the findings are particularly valuable in suggesting that it is within reach to obtain effects on generalized outcomes of linguistic comprehension. Importantly, when interpreting the results, one must bear in mind the strong stability that is observed among children in the development of language skills (Bornstein et al., 2014; Klem et al., 2015; Melby‐Lervåg and Hulme, 2012; Storch & Whitehurst, 2002). Therefore, we argue that the results indicate that the types of linguistic comprehension programs that are included in this review may be able to help improve children's language development. Simultaneously, given the modest effect sizes and the few studies with reports of follow‐up effects, we know little about whether these types of intervention programs are sufficiently influential to accelerate children's language development in the long term.

With regard to the effect of instruction on reading comprehension, it must be noted that although the synthesis of studies did not show evidence of a mean overall transfer effect from linguistic comprehension instruction to reading comprehension outcomes, there are examples of single studies that have demonstrated effects of such instruction on generalized reading comprehension outcomes (see e.g., Clarke et al., 2010). In conclusion, the review was unable to detect possible moderators that might explain divergent results across trials.

Finally, it also must be noted that the analyses of effects include trials with an intention‐to‐treat (ITT) approach, which is an accepted method for analyzing data in such trials (see e.g., Moher, Schultz, Altman, & Consort Group, 2001). The method involves estimating the effect of a trial based on how the conditions are assigned to the participants. This implies that all participants are analyzed as if they received the same amount of treatment. Consequently, because the ITT principle allows deviations from protocol and noncompliance, effect sizes are likely to be underestimated. According to Shadish, Cook, and Campbell (2002), the approach is nevertheless applicable when examining the effectiveness of a trial in field experiments, because we are interested in examining the effect of treatment as it applies to real‐world settings.

### 8.2 Implications for research

Our review detected critical aspects that can be important when designing future studies in this field:

- Evidence shows that linguistic comprehension instruction is likely to be beneficial, yet more information from RCTs is needed to ensure that there are no systematic differences between intervention groups that may affect the outcome.
- Importantly, reproducibility in experimental psychology has received much attention in recent years (Open Science Collaboration, 2015). When examining the set of studies reviewed here, it is apparent that they reach rather different conclusions regarding the effectiveness of language comprehension interventions. A few studies show large effects; however, in other studies, these effects are not replicated. In addition, in order to attain methodological thoroughness, researchers must endeavor to make their methods transparent through preregistration of protocols and in their reports, thereby making it possible for others to judge the quality of their work. This could also make it easier to explain why effects are not replicated.
- Although replicability issues can have several explanations, addressing measurement error is clearly important, since this is one factor that is likely to have a strong impact on the replicability of findings. In order to address this issue, future studies must measure effects at a construct level using latent variables. Among the studies included in this review, only a handful of studies do this.
- Notably, most of the experimental papers reviewed here do not report a correlation matrix among all variables at all timepoints. This must also be the custom for experimental studies (at least as supplemental online material), because then it will be possible to calculate an effect size *d* in future reviews that also takes into account pretest–posttest correlations. Moreover, future reviews could also then be able to employ methods such as meta‐SEM to examine effects at a construct level and controlling for measurement error.
- Finally, only a few studies have reported assessment points to examine the long‐term effect of instruction. It is important to highlight the endurance of effects when considering the effect of linguistic comprehension instruction on reading comprehension outcomes. Only a few studies in this review implemented the instructional program for a whole year or more. Programs with longer time frames and follow‐up assessments than in those included in this review must be developed in the future.

## ROLES AND RESPONSIBILITIES

There is both content and methodological expertise among the members of the review team. All the authors are working on related topics within the field of language, reading development, and intervention. Professor Monica Melby‐Lervåg and Professor Arne Lervåg have conducted several meta‐analyses and have expertise in statistical analysis. Further, the review team has experience with electronic database retrieval and have had access to library support staff when needed.

Responsibilities:

- Content: Kristin Rogde, Åste M. Hagen, Monica Melby‐Lervåg & Arne Lervåg
- Systematic review methods: Kristin Rogde, Åste M. Hagen, Monica Melby‐Lervåg & Arne Lervåg
- Statistical analysis: Kristin Rogde, Åste M. Hagen, Monica Melby‐Lervåg & Arne Lervåg
- Information retrieval: Kristin Rogde, Åste M. Hagen, Monica Melby‐Lervåg & Arne Lervåg

## SOURCES OF SUPPORT

This study was funded by the Research Council of Norway, grant number 237724.

## DECLARATIONS OF INTEREST

The authors have been involved in the development of relevant interventions and their studies are included in this review.

## PLANS FOR UPDATING THE REVIEW

A new search for studies will be conducted every fourth year by the first author (Kristin Rogde).

## Supporting information

Supplementary information
