# Supplementary material for: The effect of linguistic comprehension instruction on generalized language and reading comprehension skills: A systematic review
Source: Campbell Syst Rev. 2019 Nov 7;15(4):e1059. doi: 10.1002/cl2.1059 (PMC8356536; doi:10.1002/cl2.1059)
Supplement: Supplementary file 1 — Supplementary information [file CL2-15-e1059-s001.docx]

# Online supplement 1: Database search strategy

| Database | Hits | Search strategy |
| --- | --- | --- |
| Eric (Ovid) | 2197(631) | exp vocabulary/ or vocabular*.tw OR word knowledge.mp. [mp=abstract, title, heading word, identifiers] OR word learning.mp. [mp=abstract, title, heading word, identifiers] OR linguistic comprehension.mp. OR oral language.mp. or exp Oral Language/ OR listening comprehension.mp. or exp Listening Comprehension/ OR exp grammar/ or gramma*.tw. OR exp morphology/ or morph*.tw. OR exp syntax/ or syntax*.tw. OR syntactic*.mp. [mp=abstract, title, heading word, identifiers] OR narrative skills.mp. OR narrative comprehension.mp. OR language ability.mp. OR  language acquisition.mp. or exp Language Acquisition/ OR language comprehension.mp. OR  exp language proficiency/ or language proficiency.tw. OR exp language skills/ or language skills.tw. OR (reading adj3 comprehension).mp. [mp=abstract, title, heading word, identifiers] OR  text comprehension.mp. OR exp semantics/ or semantic*.tw. OR sentence comprehension.mp. OR  passage comprehension.mp. OR  AND exp training/ or training.tw. OR exp intervention/ or intervention.tw. OR instruction/ or reading instruction/ OR teaching.mp. OR exp learning/ or learning.tw. OR  AND exp experiments/ or experiment*.tw. OR (quasiexperiment* or quasi-experiment*).tw. OR  RCT.mp. OR randomized controlled trial*.mp. OR randomised controlled trial*.mp. OR  AND exp children/ or child*.tw. OR exp Early Adolescents/ or adolescents.mp. or exp Adolescents/ OR  elementary school student*.mp. OR exp secondary school students/ OR (secondary school student* or high school student*).tw. OR (early childhood education or elementary education or elementary secondary education or grade 1 or grade 10 or grade 11 or grade 12 or grade 2 or grade 3 or grade 4 or grade 5 or grade 6 or grade 7 or grade 8 or grade 9 or high schools or junior high schools or kindergarten or preschool education or primary education or secondary education).el.  Filters: Limit to yr="1986 -Current" |
| PsychINFO | 743(178) | exp vocabulary/ or vocabulary.tw. OR word knowledge.tw. OR word learning.tw. OR linguistic comprehension.tw. OR oral language.tw. OR listening comprehension.tw. OR exp listening comprehension/ OR exp grammar/ or (gramma* or syntax* or morph*).tw. OR narrative skills.tw. OR narrative comprehension.tw. OR language acquisition.tw. OR language comprehension.tw. OR language proficiency.tw. or exp language proficiency/ OR language skills.tw. OR exp reading/ or reading.tw. OR text comprehension.tw. OR semantic*.tw. or exp semantics/ OR sentence comprehension.tw. or exp sentence comprehension/ OR passage comprehension.tw. OR  AND intervention.mp. or exp Intervention/ OR instruct*.tw. OR teach*.tw. or exp teaching/ OR learn*.tw. or exp learning/ OR exp training/ or training.tw. OR AND quasiexperiment*.tw. or quasi-experiment*/ OR RCT.tw. OR randomi?ed controlled trial*.tw. OR (random* or sham or placebo*).ti,ab,hw. OR ((singl* or doubl*) adj (blind* or dumm* or mask*)).ti,ab,hw. OR ((tripl* or trebl*) adj (blind* or dumm* or mask*)).ti,ab,hw. OR AND adolescen*.tw. OR elementary school student*.tw. or exp elementary school students/ OR primary school student*.tw. or exp primary school students/ OR (secondary school student* or high school student*).tw. OR exp Preschool Students/ or preschool*.tw. or exp Preschool Education/ OR kindergarten.tw. or exp Kindergartens/ OR exp Kindergartens/ or exp Kindergarten Students/ or kindergartner*.tw.  Filters:  Limit to yr="1986 -Current"  “100”.ag  “160”.ag  “180”.ag.  “200”.ag. |

| Scopus Science Direct | 969(2) | ( title-abs-key( (vocabular* or "word knowledge" or "word learning" or "linguistic comprehension" or "oral language" or "listening comprehension" or "language proficiency" or "languagecomprehension"or "language skills" or "language abilities" or "language acquisition" or gramma* or syntax or syntactic* or semantic* or morph* or "narrative skills" or "narrative comprehension" or reading w/15 comprehension or "text comprehension" or "sentence comprehension" or "passage comprehension"))) and (title-abs-key ((preschool* or kindergart* or school* or "day care*" or adolescen* or student*or "high school" or elementary*or primary* or secondary* ) ) ) and ( title-abs-key ( ( intervention or training or instruct*or learn* or teach*))) and (title-abs-key ( ( experiment* or "quasi-experiment*" or quasiexperiment or rct or "randomi?ed controlled trial*"))) and (exclude (subjarea , "envi" ) or exclude(subjarea, "deci") or exclude ( subjarea , "eart" ) or exclude ( subjarea , "chem" ) or exclude(subjarea , "mate" ) or exclude( subjarea , "phys") or exclude(subjarea,"undefined"))  Filters: Limit to years: 1986-current |
| --- | --- | --- |
| Proquest Digital | 213(23) | ((Intervention OR Training OR Instruct* OR SU("Reading Instruction" OR "Second Language Reading Instruction") OR SU("Vocabulary Instruction") OR SU("Oral Language Instruction") OR SU("Linguistics Instruction") OR SU("Grammar Instruction") OR SU("Transfer (Learning)") OR SU("Second Language Vocabulary Learning" OR "Vocabulary Learning") OR Teach*) AND (SU("Vocabulary") OR ("word knowledge") OR ("word learning") OR ("Linguistic comprehension") OR SU("Linguistic Competence") OR SU("Oral Language") OR SU("Listening Comprehension") OR ("language proficiency") OR ("language comprehension") OR ("Language skills") OR ("language abilities") OR ("language acquisition") OR (Gramma*) OR (Syntax) OR SU("Syntactic Processing") OR (Semantic*) OR SU("Morphological Processing") OR SU("Affixes" OR "Morphemes" OR "Prefixes" OR "Roots (Morphology)" OR "Suffixes") OR SU("Morphology" OR "Semantics Morphology Relationship") OR SU("Morphological Analysis" OR "Segmentation") OR ("narrative skills") OR ("narrative comprehension") OR SU("Reading Comprehension") OR (Reading NEAR/15 comprehension) OR ("text comprehension") OR ("sentence comprehension") OR ("Passage comprehension")) AND (SU("Elementary School Students") OR SU("Elementary Education") OR SU("High School Students" OR "Junior High School Students" OR "Secondary School Students") OR SU("Junior High School Students") OR SU("Junior High School Education") OR SU("High School Students") OR SU("Preschool Children") OR SU("Preschool Education") OR SU("Kindergarten")) AND ((Experiment*) OR ("quasi-Experiment*") OR (quasiexperiment) OR RCT OR ("randomi?ed controlled trial")))  Filters: Limit to years: 1986-current |
| Linguistics and language Behaviour Abstracts (LLBA) | 725(234) | ((Intervention OR Training OR Instruct* OR SU("Reading Instruction" OR "Second Language Reading Instruction") OR SU("Vocabulary Instruction") OR SU("Oral Language Instruction") OR SU("Linguistics Instruction") OR SU("Grammar Instruction") OR SU("Transfer (Learning)") OR SU("Second Language Vocabulary Learning" OR "Vocabulary Learning") OR Teach*) AND (SU("Vocabulary") OR ("word knowledge") OR ("word learning") OR ("Linguistic comprehension") OR SU("Linguistic Competence") OR SU("Oral Language") OR SU("Listening Comprehension") OR ("language proficiency") OR ("language comprehension") OR ("Language skills") OR ("language abilities") OR ("language acquisition") OR (Gramma*) OR (Syntax) OR SU("Syntactic Processing") OR (Semantic*) OR SU("Morphological Processing") OR SU("Affixes" OR "Morphemes" OR "Prefixes" OR "Roots (Morphology)" OR "Suffixes") OR SU("Morphology" OR "Semantics Morphology Relationship") OR SU("Morphological Analysis" OR "Segmentation") OR ("narrative skills") OR ("narrative comprehension") OR SU("Reading Comprehension") OR (Reading NEAR/15 comprehension) OR ("text comprehension") OR ("sentence comprehension") OR ("Passage comprehension")) AND (SU("Elementary School Students") OR SU("Elementary Education") OR SU("High School Students" OR "Junior High School Students" OR "Secondary School Students") OR SU("Junior High School Students") OR SU("Junior High School Education") OR SU("High School Students") OR SU("Preschool Children") OR SU("Preschool Education") OR SU("Kindergarten")) AND ((Experiment*) OR ("quasi-Experiment*") OR (quasiexperiment) OR RCT OR ("randomi?ed controlled trial"))) AND la.exact("English") AND pd(19860101-20160302)  Filters: Limit to years: 1986-current |
| Bielefeld Academic Search Engine (BASE | * | Search 1: (vocabular or language) AND (instruction or training) AND RCT Search 2: (vocabulary or language) AND (instruction or training) AND quasi-experiment Search 3: (“linguistic comprehension”) AND instruction  Search 4: (“language comprehension”) AND (instruction or training)  Search 5: (tit: vocabular) AND (tit:randomized)  Filters: “1986-current”  *The 500 most relevant references (the first 100 for each search string) were included for further screening. |
| *Google Scholar* | * | Search 1: (vocabular or language) AND (instruction or training) AND RCT  Search 2: (vocabulary or language) AND (instruction or training) AND quasi-experiment Search 3: (“linguistic comprehension”) AND instruction  Search 4: (“language comprehension”) AND (instruction or training)  Search 5: (tit: vocabular) AND (tit:randomized)  Filters: “1986-current  *The 500 most relevant references (the first 100 for each search string) were included for further screening. |
| *Open Grey* | 27(0) | (Preschool* OR Kindergart* OR School* OR "day care*" OR elementary school OR primary school OR secondary school OR early childhood education) AND(Experiment* OR "quasi-Experiment*" OR quasiexperiment OR RCT OR "randomi?ed controlled trial")AND (intervention OR training OR instruct* OR learn* OR teach*) AND (vocabulary OR "Word knowledge" OR "Word Learning" OR "linguistic comprehension" OR "oral Language" OR "listening comprehension" OR "Language proficiency" OR "Language comprehension" OR "Language skills" OR "Language abilities" OR "Language acquisition" OR gramma* OR syntax OR syntactic* OR semantic* OR morph* OR "narrative skills" OR "narrative comprehension" OR Reading NEAR/15 comprehension OR "text comprehension" OR "sentence comprehension" OR "passage comprehension") |
| *ISI Web of Science* | 1139(532) | #1  (TS= (intervention OR training OR instruct* OR learn* OR teach*)) AND LANGUAGE: (English)  DocType=All document types; Language=All languages;  #2  (TS= (Preschool* OR Kindergart* OR School* OR "day care*")) AND LANGUAGE: (English)  DocType=All document types; Language=All languages;  #3  (TS= (Experiment* OR "quasi-Experiment*" OR quasiexperiment OR RCT OR "randomi?ed controlled trial")) AND LANGUAGE: (English)  DocType=All document types; Language=All languages;  #4  (TS= (vocabulary OR "Word knowledge" OR "Word Learning" OR "linguistic comprehension" OR "oral Language" OR "listening comprehension" OR "Language proficiency" OR "Language comprehension" OR "Language skills" OR "Language abilities" OR "Language acquisition" OR gramma* OR syntax OR syntactic* OR semantic* OR morph* OR "narrative skills" OR "narrative comprehension" OR Reading NEAR/15 comprehension OR "text comprehension" OR "sentence comprehension" OR "passage comprehension")) AND LANGUAGE: (English)  DocType=All document types; Language=All languages;  #5  #4 AND #3 AND #2 AND #1  DocType=All document types; Language=All languages;  # 6 (filters language and years)  #4 AND #3 AND #2 AND #1  Refined by: LANGUAGES: ( ENGLISH )  Indexes=SSCI, A&HCI, CPCI-S, CPCI-SSH, ESCI Timespan=1986-2016  #7 (filters excluding disiplines)  #4 AND #3 AND #2 AND #1  Refined by: LANGUAGES: ( ENGLISH ) AND [excluding] WEB OF SCIENCE CATEGORIES: ( CLINICAL NEUROLOGY OR MECHANICS OR MATHEMATICAL COMPUTATIONAL BIOLOGY OR MATHEMATICS OR AUTOMATION CONTROL SYSTEMS OR GEOSCIENCES MULTIDISCIPLINARY OR REHABILITATION OR ANTHROPOLOGY OR GEOGRAPHY OR FAMILY STUDIES OR COMPUTER SCIENCE HARDWARE ARCHITECTURE OR ENGINEERING MECHANICAL OR ECONOMICS OR ROBOTICS OR BIOTECHNOLOGY APPLIED MICROBIOLOGY OR COMPUTER SCIENCE INTERDISCIPLINARY APPLICATIONS OR REMOTE SENSING OR ARCHITECTURE OR COMPUTER SCIENCE ARTIFICIAL INTELLIGENCE OR PSYCHOLOGY BIOLOGICAL OR URBAN STUDIES OR COMPUTER SCIENCE THEORY METHODS OR PHYSIOLOGY OR SUBSTANCE ABUSE OR COMPUTER SCIENCE INFORMATION SYSTEMS OR OPTICS OR SPORT SCIENCES OR OPERATIONS RESEARCH MANAGEMENT SCIENCE OR SOCIAL WORK OR NURSING OR SOCIAL SCIENCES MATHEMATICAL METHODS OR ENGINEERING ELECTRICAL ELECTRONIC OR MATERIALS SCIENCE MULTIDISCIPLINARY OR PRIMARY HEALTH CARE OR LITERATURE ROMANCE OR PLANT SCIENCES OR ERGONOMICS OR PHYSICS CONDENSED MATTER OR ENVIRONMENTAL STUDIES OR PHYSICS APPLIED OR NEUROSCIENCES OR ENGINEERING MULTIDISCIPLINARY OR OTORHINOLARYNGOLOGY OR COMPUTER SCIENCE SOFTWARE ENGINEERING OR ENGINEERING INDUSTRIAL OR ORTHOPEDICS OR PEDIATRICS OR BUSINESS OR NUTRITION DIETETICS OR INFORMATION SCIENCE LIBRARY SCIENCE OR BIOCHEMICAL RESEARCH METHODS OR NEUROIMAGING OR COMPUTER SCIENCE CYBERNETICS OR SURGERY OR LITERARY THEORY CRITICISM OR RADIOLOGY NUCLEAR MEDICINE MEDICAL IMAGING OR HOSPITALITY LEISURE SPORT TOURISM OR PSYCHOLOGY MATHEMATICAL OR GERONTOLOGY OR TELECOMMUNICATIONS OR OBSTETRICS GYNECOLOGY OR GERIATRICS GERONTOLOGY OR PSYCHIATRY OR MUSIC OR GASTROENTEROLOGY HEPATOLOGY OR MEDICINE GENERAL INTERNAL OR MEDICAL INFORMATICS OR ENDOCRINOLOGY METABOLISM OR MANAGEMENT OR HUMANITIES MULTIDISCIPLINARY OR CONSTRUCTION BUILDING TECHNOLOGY OR ACOUSTICS OR ENGINEERING BIOMEDICAL OR CHEMISTRY MULTIDISCIPLINARY OR IMAGING SCIENCE PHOTOGRAPHIC TECHNOLOGY OR STATISTICS PROBABILITY OR CELL BIOLOGY OR PHARMACOLOGY PHARMACY OR BIOLOGY OR MEDICINE RESEARCH EXPERIMENTAL OR ZOOLOGY )  Indexes=SSCI, A&HCI, CPCI-S, CPCI-SSH, ESCI Timespan=1986-2016 |

*Hits*=Original search (follow-up search)

# Online Supplement 2: Excluded studies

Exemplary excluded studies are presented below for different categories of exclusion (although each could have been excluded for several reasons).

Studies excluded because of the type of outcome measures

Studies that only included proximal outcome measures inherent to the treatment or that did not report post-test data for generalized linguistic comprehension skills were not eligible to be included in the review (e.g., Arthur & Davis, 2016). Similarly, if a study described a considerable overlap between words taught in an intervention and an outcome measure that was originally considered to measure general language skills (e.g., standardized tests), the study was excluded, as long as it reported no other general language outcomes (e.g., Fehr, 2011). Further, studies that only included an assessment of general language at pretest (typically to determine whether the treatment and control groups were equivalent in terms of their language proficiency) and not at posttest were not considered eligible (e.g., Beck & McKeown, 2007; Chlapana & Tafa, 2014; Filippinin, Gerber, & Leafstedt, 2012).

Studies excluded because of type of comparison conditions

Studies in which the control group received a different type of instructional method for linguistic comprehension training were not eligible. This eliminated studies of vocabulary training that focused on comparing different methods of vocabulary instruction (e.g., Curtis, 2008; Nash & Snowling 2006). Similarly, we excluded studies in which the comparison groups could not be interpreted as a business-as-usual control because of some type of additional language- or literacy-related instruction (Motsch & Marks, 2014; Coyne et al., 2004; Bowyer-Crane et al., 2008; Adlof, McLeod & Leftwich, 2014; Roberts & Neal, 2004). We also excluded studies that reported that the comparison group was exposed to the same set of books as the intervention group (e.g., Stevens, Van Meter, & Warcholak, 2010).

Studies excluded because of the content in the intervention program

We excluded studies of professional teacher programs that targeted children’s language skills indirectly (e.g., Bowne, Yoshikawa, & Snow, 2016; Cabell et al., 2011; Gersten, Dimino, Jayanthi, Kim, & Santoro, 2010; Neuman, Pinkham, & Kaefer, 2015; Snow et al., 2014; Wasik & Bond, 2006; Wasik & Hindman, 2011; Yoshikawa et al., 2015). In contrast to the studies included in this review, these studies define their focus of instruction on teachers’ knowledge of language development, and they involve a more extensive and intensive coaching of teachers. When screening the literature, it became evident that there is a large number of intervention studies that target children’s reading comprehension skills with a variety of instruction features and amount of language comprehension instruction. Therefore, we decided that studies had to be defined as a vocabulary, comprehension-based or oral language intervention program with at least 50% amount of focus of instruction in the program. Therefore, only studies with the main aim of targeting linguistic comprehension instruction were eligible. We excluded studies that mainly targeted children’s skills of monitoring their reading process through strategy instruction or had up to 50% of the time devoted to decoding instruction. Thus, consequently, studies with different types of reading intervention programs are excluded. This could be intervention programs characterized as code-related reading instruction, motivational instruction, multicomponent reading instruction, reading comprehension strategy instruction, or collaborative strategic reading (e.g., Droop et al., 2016; Kirk & Gillon, 2009; Solìs, Vaughn & Scammacca, 2015; Vadasy et al., 2008; Kim et al., 2006; Vaughn et al., 2011). In addition, as described in the section on eligible criteria, studies that included elements of instruction in children’s home environment were not included (Zevenbergen, Whitehurst, & Zevenbergen, 2003; Lee & Pring, 2016).

Studies excluded because of a change in design characteristics

One study met our inclusion criteria but was excluded before conducting the analyses due to changes in the study design after the onset of our study. The pre-post-controlled experiment by Appel & Vermeer (1998) reported that one of the experimental schools spent from 2–4 weeks on the program but did not follow the program after this. Therefore, this school was considered a comparison school instead of an experimental school, as originally planned.

Studies excluded because of design

Even though regression discontinuity design is typically considered a quasi-experimental study, studies using this design were excluded (e.g., Dyson, Solity, Best, & Hulme, 2018) because this was not specified in the protocol and review onset. Another type of studies that were excluded were studies that used a response to instruction approach (Lonigan & Phillips, 2016).

# Online Supplement 3: Risk of bias Assessment

| Study* (design) |  | Selection bias | Performance bias | Detection bias | Attrition bias | Reporting bias |
| --- | --- | --- | --- | --- | --- | --- |
| Apthorp, 2006 | **Judgement** | *High risk* | *High risk* | *Unclear risk* | *High risk* | *High risk* |
|  | **Description** | “Random assignment of teachers and classrooms to treatment or control conditions within participating schools.” Site A: 4 schools; Site B: 3 schools. | Not possible | Not reported | Attrition rate site A: 8,3% and 4,1% for treatment and control, respectively. “In site B, the attrition rates were 8,6 and 10,5% for treatment and control, respectively”.  “Across both sites, data are reported for 299 children“. Posttest Site A: n=124; Site B: n= 144) | Missing information about effect size in Site B on the oral vocabulary task. |
| Apthorp et al., 2012 | **Judgment** | *Low risk* | *High risk* | *Unclear risk* | *Low risk* | *Low risk* |
|  | **Description** | “44 Schools participated..”. “The schools were randomly assigned to either the primary or intermediate grade treatment group.” | Not possible | “All student assessments were group administered by trained examiners who were not teachers of the students being assessed.” Unclear if scoring were blinded or not. | Similar attrition rates, not considered to contribute to bias | No indication of reporting bias |
| Block & Mangieri 2006 | **Judgment** | *Low risk* | *High risk* | *Unclear risk* | *Unclear risk* | *Low risk* |
|  | **Description** | The classrooms of the students in the three elementary schools and one middle school were randomly assigned to experimental or control groups by site coordinators prior to the study. | Not possible | Not reported | Unclear reporting | No indication of reporting bias |
| Brinchmann et al., 2015 | **Judgment** | *High risk* | *High risk* | *Unclear risk* | *Unclear risk* | *Low risk* |
|  | **Description** | Nonrandom sampling procedure | Not possible | Not reported if the assessment are blinded or not | Not reported | No indication of reporting bias |
| Cable, 2007 (QE) | **Judgment** | *High risk* | *High risk* | *Low risk* | *Low risk* | *Low risk* |
|  | **Description** | “Of the 180 screened, 57 met the criteria for inclusion in the study and 37 students returned signed parent consent forms. Students who returned consent forms were randomly assigned to treatment and comparison conditions”. “After randomization, the investigator examined the distribution of students who were designated by the school as Limited English Proficient (LEP) in each group and found that the control group had a higher proportion of LEP students. All LEP students were redistributed evenly between groups and other students were then  randomly chosen and re-assigned to make the group numbers equivalent.” | Not possible | “Screening, pretest and posttest measures were administered by licensed speech-language pathologists and supervised graduate students in speech-language pathology who were unaware of the treatment conditions” | The same number of participants are reported at the two time-points (pre- and posttest) | No indication of reporting bias |
| Clarke et al., 2010 | **Judgment** | *Low risk* | *High risk* | *Unclear risk* | *Low risk* | *Low risk* |
|  | **Description** | Twenty schools took part in the study. Each school employed one assistant to implement the intervention programs. Eight participants within each school were randomly assigned to the four conditions. | Not possible | Not reported if the assessment are blinded or not | Low attrition rates | No indication of reporting bias |
| Coyne et al., 2010 | **Judgment** | *High risk* | *High risk* | *Unclear risk* | *Low risk* | *Low risk* |
|  | **Description** | Three schools: Intact classrooms were assigned to either treatment or control conditions. School A: 2 classes were assigned to implement the experimental intervention, 1 classroom served as control.  School B: One classroom implemented the intervention and one served as control. School C: All students across the three classrooms were randomly assigned at the student level to receive intervention or serve as a control. | Not possible | No information about blinding procedures. | “One student did not participate because of teacher recommendation, four participants moved out of the district, two participants were not adm. Posttest measures..” (Crevecoeur, 2014) | No indication of reporting bias |
| Crain-Thoreson et al., 1999 | **Judgment** | *High risk* | *High risk* | *Low risk* | *Low risk* | *Low risk* |
|  | **Description** | Based on the children’s pretest scores, triads of children with similar receptive vocabulary scores were formed. “Children within each triad were randomly assigned…” Staff/practice n=13; Staff/control: n=11). Final group size: Treatment: 13; Control: n=9. | Not possible. “At two schools, one staff member read to all the children in both the staff/practice and the Staff/control groups. | Blinded | Two children in the control group did not complete the study. | No indication of reporting bias |
| Dockrell et al., 2010 | **Judgment** | *High risk* | *High risk* | *Low risk* | *Low risk* | *Low risk* |
|  | **Description** | No randomization | Not possible | Assessors were blind to the intervention. | Equal attrition between groups | No indication of reporting bias |
| Farver et al., 2009 | **Judgment** | *Low risk* | *High risk* | *Low risk* | *Low risk* | *Low risk* |
|  | **Description** | “Ninety-six children were randomly assigned balanced for gender. Children were assigned to conditions within classrooms to avoid problems with nesting”. | Not possible | Blinded | Low attrition rate | No indication of reporting bias |
| Fricke et al., 2013 | **Judgment** | *Low risk* | *High risk* | *Unclear risk* | *Low risk* | *Low risk* |
|  | **Description** | Random assignment to treatment and control groups | Not possible | Not reported | Children lost intervention, posttest: N=7; Control: N=6 | No indication of reporting bias |
| Fricke et al., 2017 | **Judgment** | *Low risk* | *High risk* | *Low risk* | *Low risk* | *Low risk* |
|  | **Description** | Random assignment to treatment and control groups | Not possible | “All testers were blind to group allocation” | Missing data are considered to be missing completely at random. | No indication of reporting bias |
| Gonzalez et al., 2010 | **Judgment** | *Low risk* | *High risk* | *Unclear risk* | *Low risk* | *Low risk* |
|  | **Description** | Teachers were randomly assigned to intervention (n=13) or practice as usual (n=8) condition. “Twenty-one teachers and 148 children from their classrooms (n=28) were randomly assigned at the class level to either the Words of Oral Reading and Language Development intervention or a practice as usual condition.” Students were nested within teacher. | Not possible | No information about blinded assessment | “There was no evidence of differential attrition between intervention and comparison conditions” | No indication of reporting bias |
| Hagen et al., 2017 | **Judgment** | *Low risk* | *High risk* | *Low risk* | *Low risk* | *Low risk* |
|  | **Description** | This is a cluster-randomized trial, randomization has been conducted at the classroom level. Because there is a high number of classrooms (n=150) and participants (n=301), this study is rated as low risk on the selection bias scale even though the randomization is not at the individual level. | Not possible | Blinded assessment | “37 children were lost from the trial by the end of the study”. “..the children with missing data were not significantly different from the children without missing data”. | No indication of reporting bias |
| Haley et al., 2017 | **Judgment** | *Low risk* | *High risk* | *Low risk* | *Low risk* | *Low risk* |
|  | **Description** | Individual randomization | Blinding not possible | All testers were blind to group membership with the exception of the first author who conducted on-site tutorials where she observed an intervention session taking place, thereby gaining awareness of group membership before posttesting occurred. | 1 lost in treatment group; 4 lost in the control group | No indication of reporting bias |
| Johanson et al., 2016 | **Judgment** | *High risk* | *High risk* | *Low risk* | *High risk* | *Low risk* |
|  | **Description** | Teachers signed up, 11 in total; not wholly random selection | Not possible | Blinded assessment | One teacher dropped out after random selection | No indication of publication bias |
| Justice et al., 2008 | **Judgment** | *Low risk* | *High risk* | *Low risk* | *High risk* | *Low risk* |
|  | **Description** | Fourteen preschool teachers were randomly assigned to 2 conditions. | Not possible | “Research assistants were blind to study conditions when transcribing and coding child language samples” | “There were some missing data for fall and spring expressive language scores” | No indication of reporting bias |
| Justice et al., 2010 | **Judgment** | *High risk* | *High risk* | *Low risk* | *High risk* | *Low risk* |
|  | **Description** | 14 schools in four districts participated in the program evaluation. 11 Teachers served as an experimental group (RIA) and 9 teachers served as control. Teachers were provided the opportunity to participate. 18 teachers provided informed consent. Project resources mandated that data could be collected from 11 classrooms – random selection of the 18 teachers to participate. The 11 experimental teachers in this study were part of the pool of 18 teachers who had implemented and evaluated draft RIA materials in the previous year. | Not possible | “After test administrations were completed, scoring of measures was completed by undergraduate research assistants…and who were blind to study conditions of the participants.” | Total 137 children: 66 treatment; 71 control. At pretest N= 66 for the treatment group and 58 in the comparison group.  Posttest treatment group: 13 participants are lost.  Posttest control group: 1 participant is lost. | No indication of reporting bias |
| Kelley et al., 2015 | **Judgment** | *High risk* | *High risk* | *Low risk* | *Low risk* | *Low risk* |
|  | **Description** | “A total of 18 children, 6 in each of three classrooms, were randomly assigned to treatment or comparison condition.” Small sample size and unclear information about how these 18 children have been selected. | “A total of 18 children, 6 in each of three classrooms, were randomly assigned to treatment or comparison condition.” Small sample size and unclear information about how these 18 children have been selected. | “Scorers were blinded to participants and assessment points”. | No sign of attrition | No indication of reporting bias |
| Lawrence et al., 2015 | **Judgment** | *Low risk* | *High risk* | *Unclear risk* | *Low risk* | *Low risk* |
|  | **Description** | “Twenty-eight schools in two districts participated in this randomized trial.” “Once the schools had agreed to participate, we randomized schools to treatment conditions. | Not possible | No information about blinded assessment | Large attrition rates are reported due to logistical difficulties. Pretest differences between students who did and did not complete both waves of academic vocabulary testing showed no differences. | No indication of reporting bias |
| Lawrence et al., 2017 | **Judgment** | *Low risk* | *High risk* | *Unclear risk* | *High risk* | *Low risk* |
|  | **Description** | “Forty-four middle schools in three urban districts were randomly assigned to treatment or control conditions” | Not possible | No information about blinded assessment or not | “Two schools assigned to control and one school assigned to treatment dropped out of the study and did not provide data”. | No indication of reporting bias |
| Lesaux et al., 2010 | **Judgment** | *High risk* | *High risk* | *Unclear risk* | *Low risk* | *Low risk* |
|  | **Description** | No randomization | Not possible to blind participants or tutors | Not possible to blind participants or tutors | 123 (6%) missing posttest data largely due to moving. Missing 417 students (20%) on one or more pretests at one school, the authors report no difference in pretest scores between groups. | No indication of reporting bias |
| Lesaux et al., 2014 | **Judgment** | *Low risk* | *High risk* | *Unclear risk* | *High risk* | *Low risk* |
|  | **Description** | Teachers (n=50) were randomly assigned to treatment or control group. | Not possible to blind participants or tutors | No report of blinded assessment or not | No report of blinded assessment or not | No indication of reporting bias |
| Lonigan et al., 1998 | **Judgment** | *Low risk* | *High risk* | *Low risk* | *Low risk* | *Low risk* |
|  | **Description** | After consent and pretest, children were randomly assigned within classrooms to one of four experimental conditions. (4 Child care centers). Reassignment in one of the centers to achieve balance based on pretest scores. | Not possible | Blinded (most of the time) | 23 out of 114 lost to post test. A comparison between the 91 children who completed the posttest and the 23 who could not be posttested indicated that they did not differ on any of the pretest variables. | No indication of reporting bias |
| Lonigan et al., 1999 | **Judgment** | *Low risk* | *High risk* | *Low risk* | *Low risk* | *Low risk* |
|  | **Description** | “All children were randomly assigned within centers to one of three experimental conditions”. | Not possible | “The assessors were familiar with th design of the study but were not aware of a child´s assignment to condition”. | “Of the 110 children initially recruited, 15 left their center before the study ended, thus our sample was comprised of 95 children who completed both pretest and posttest. There were no differences on the standardized measures used at pretest between the 95 children in our sample and the 15 who could not be posttested”. | No indication of reporting bias |
| Lonigan et al., 2013 | **Judgment** | *Low risk* | *High risk* | *Low risk* | *Low risk* | *Low risk* |
|  | **Description** | Random assignment of children within schools | Not possible | “Trained research assistants, who were blind to group assignments and study hypotheses, testes children..” | 89% completed some posttest measures, but no difference at pretest | No indication of reporting bias |
| Murphy et al., 2016 | ***Judgment*** | *High risk* | *High risk* | *High risk* | *High risk* | *High risk* |
|  | **Description** | In total 4 schools are represented in the study. “Two schools were randomly allocated to the intervention group and the other two schools to the control group”. | Not possible to blind | The researchers were blinded to the performance of the student at the previous assessment point but had knowledge of whether they had or had not received the intervention. | 11 students were excluded from the analysis because they were unavailable for testing at any point. Reasons or group affiliation for these students are not reported. | “Students who were unavailable for testing at any point were excluded from the analysis (N = 11)”. “Students having English as an additional language (N = 31) were included in the study, but not in the analysis”. |
| Neuman et al., 2011 | **Judgment** | *Low risk* | *High risk* | *Unclear risk* | *Unclear risk* | *Low risk* |
|  | **Description** | Six schools were randomly assigned to treatment and six to the control group. | Not possible | No report about blinded assessment or not | At the delayed posttet (six months later), approximately one third of the students had continued in the program. Unclear information about attrition rates for the immediate posttest time-point. | No indication of reporting bias |
| Nielsen et al., 2012 | **Judgment** | *High risk* | *High risk* | *Unclear risk* | *Low risk* | *Low risk* |
|  | **Description** | Children scoring low on language tests are included in the study. Assignment to groups are based on matching. | Not possible | Not reported | “While we began with  30 participants, after the first 2 weeks of intervention, one intervention student was dismissed from the study due to severe behavior problems in the classroom and the intervention, and a student in the control group moved out of the district.” | No indication of reporting bias |
| Phillips et al., 2016 | **Judgment** | *Low risk* | *High risk* | *Low risk* | *Low risk* | *Low risk* |
|  | **Description** | The 82 selected children were randomly assigned within school (and classroom where possible) to the treatment or control conditions. | Not possible | All posttesting was conducted by assessors who were blind to children’s treatment status and who had had no prior contact with the them. Pre- and posttest data were anonymized and pooled prior to double scoring by blind scorers. | An attrition rate of 6.1% (considered to be low). The eight children missing at posttesting were divided equally between the treatment and control group. | No indication of reporting bias |
| Pollard-Durodola et al., 2011 | **Judgment** | *Low risk* | *High risk* | *Unclear risk* | *Low risk* | *Low risk* |
|  | **Description** | Children were stratified by classroom and randomly assigned to one of two shared book-reading conditions. “The researchers initially chose teachers from six schools across two school districts and randomly assigned them…” | Not possible | No report of blinding | Of the 148 students originally in the study, 23 (16%) dropped out before the study was completed. One teacher dropped out before the intervention began, another opted not to participate during the intervention, and students typically dropped out because their families moved or because they withdrew from school during the school year.” Analysis showed nonsignificant difference in attrition rates between groups. | No indication of reporting bias |
| Proctor et al., 2011 | **Judgment** | *High risk* | *High risk* | *Unclear risk* | *Unclear risk* | *Low risk* |
|  | **Description** | “..129 students from 6 classrooms in 3 of the 4 schools received the ICON intervention and were compared with 111 students in 6 classrooms in 2 of the 4 schools..” No randomization to conditions. | Not possible | No information about blinded assessment or not. | No report of attrition or N status at the posttest assessment | No indication of any reporting bias. |
| Rogde et al., 2016 | **Judgment** | *Low risk* | *High risk* | *Low risk* | *Low risk* | *Low risk* |
|  | **Description** | Randomization; individual (N=115) | Not possible | Research assistants conducting the assessments were blinded to the children’s group affiliation | Small attrition due to moving schools | No indication of reporting bias |
| Scaefer et al., (unpublished) | **Judgment** | *Low risk* | *High risk* | *Unclear risk* | *Low risk* | *Low risk* |
|  | **Description** | “Eighty children were randomly allocated to receive an 18-week oral language intervention…”. | Not possible | No report of blinding | Report low attrition rate | No indication of reporting bias |
| Silverman et al., 2013 | **Judgment** | *High risk* | *High risk* | *Unclear risk* | *High risk* | *Low risk* |
|  | **Description** | Classrooms were randomly assigned to a condition, but differences were found between groups. | Not possible | No report of blinding | 16% (n=60) were absent on at least one of the four testing days | No indication of reporting bias |
| Simmons et al., 2010 | **Judgment** | *Low risk* | *High risk* | *Unclear risk* | *Low risk* | *Low risk* |
|  | **Description** | Schools (n=15) in Districts 1 and 2 were matched on the previous year’s reading achievement performance on the TAKS | Not possible | No reporting of testing procedures were blinded or not | The primary reason for attrition is moving to another school. No difference between the different groups, assuming that the changes are random. | No indication of reporting bias |
| Spencer et al., 2014 | **Judgment** | *High risk* | *High risk* | *Low risk* | *Low risk* | *Low risk* |
|  | **Description** | After matching the groups, they were randomly assigned to treatment and comparison conditions. | Not possible | Research assistants were blind to group assignment. | Low attrition rate, equal between groups. | No indication of reporting bias |
| Styles et al., 2015 | **Judgment** | *Low risk* | *High risk* | *High risk* | *Low risk* | *Low risk* |
|  | **Description** | Pupils were deemed eligible for the study and were consented to be randomized to the intervention or control groups. Randomized at pupil level, stratified by school. ”Randomized at pupil level to two groups – within the same school”. | Not possible | “…no significant difference between groups for the data after attrition”. | “…no significant difference between groups for the data after attrition”. | No indication of reporting bias |
| Vadasy et al., 2015 | **Judgment** | *High risk* | *High risk* | *Low risk* | *High risk* | *Low risk* |
|  | **Description** | Teachers were randomly assigned to treatment or control prior to student pretesting. All students in each participating teacher´s classroom were invited to participate each year. | Not possible | Trained testers unaware of experimental group assignment | 3% attrition (22 treatment and 22 control). Randomization has been conducted before consent has been received. | No information that assumes reporting bias. |
| Valdez-Menchaca et al., 1992 | **Judgment** | *High risk* | *High risk* | *Low risk* | *Low risk* | *Low risk* |
|  | **Description** | Matched-pair experimental design. Pairs of children were matched as nearly as possible on the basis of mean length of utterance, family income, level of maternal education, family size, and gender. | Not possible | The trained assistant was unaware of the children’s experimental status. | No attrition are reported. Because this is a matched experiments over a relatively short period of time, low attrition are assumed. | No indication of reporting bias |
| Van Kleeck et al., 2006 | **Judgment** | *High risk* | *High risk* | *Low risk* | *Low risk* | *Low risk* |
|  | **Description** | “The African American and the Caucasian groups of children were divided randomly into treatment (n=15) and control groups (n=15)”. | Not possible | Some of the research assistants who administered the posttest for the study were not blind to the children’s group assignment, even though the scorer was not aware of this. | No report of attrition rates. One can assume that there is no severe attrition based on the length of the intervention and since nothing has been reported about this. | No indication of reporting bias |
| Wasik et al., 2011 | **Judgment** | *High risk* | *Low risk* | *Unclear risk* | *Low risk* | *Low risk* |
|  | **Description** | “Two teachers were randomly assigned to the intervention condition, and two were assigned to the control condition”. (Represented by one preschool) | Not possible | Not reported | “Initially, there were 64 children  in the intervention group and 63 in the control group. Six children transferred from the school, leaving 61 in the intervention and 60 in the control group.” | No indication of reporting bias |
| Whitehurst et al., 1994 | **Judgment** | *High risk* | *High risk* | *Low risk* | *Low risk* | *Low risk* |
|  | **Description** | 73 subjects were assigned randomly within classrooms to 1 of 3 conditions. Reassignment was necessary in one classroom to achieve parity in pretest scores across the groups. | Not possible | “The assessors knew the design and hypotheses of the study but were not familiar with a child´s assignment to condition at the time an assessment was conducted”. | Small attrition rate from pretest to posttest. (Larger at follow-up.) | No indication of reporting bias |

# Online supplement 4: P-Curve Disclosure Table

**STUDY SELECTION RULES**

- Interventions that aim to improve linguistic comprehension and/or reading comprehension through linguistic comprehension tasks.
- Interventions that explicitly test a hypothesis concerning generalized or standardized outcomes.
- Participants approximate age 4-16
- Studies published between 1986 and 2016

| Study | Prediction of interest to researcher (quote from paper) | Study design – reported information | Key statistical result | Results quoted from paper | Results with precise p-value | Robustness results |
| --- | --- | --- | --- | --- | --- | --- |
| *Apthorp, 2006* | The challenge for educators to reduce or prevent large  achievement gaps is getting children onto the right trajectory  of vocabulary growth and reading development and helping them maintain that growth. | RCT (cluster) | Multivariate analysis of variance (MANOVA)  with condition as the between-subjects  factor  and univariate ANOVAs | In Site A, the MANOVA conducted on reading vocabulary  and comprehension revealed a significant condition effect, *F*(2, 121) = 8.13, *p* < .001, *ES* = 0.73. The follow-up  univariate ANOVAs showed a significant effect of condition on reading vocabulary, *F*(1, 122) = 9.123, *p* < .005, *ES*  = 0.55, but not on reading comprehension, *F*(1, 128) = –0.067, *ES* = –0.06. | *F*(2, 121) = 8.13, *p* < .001 | *F*(1, 122) = 9.123, *p* < .005, |
| *Apthorp et al., 2012* | We hypothesized that continued exposure to EOR:  V for 2 consecutive years would produce measurable changes in student performance on  distal measures of standardized tests of vocabulary and passage comprehension | RCT (cluster within blocks – a four-block design) | Two-level hierarchical linear model with students  nested in schools | In Year 2, EOR: V had no statistically significant impact on the standardized measures of vocabulary or passage comprehension in either the primary or intermediate grades (see  Table 10). | Excluded due to no significant effects on generalized measures |  |
| *Block & Mangieri, 2006* | (…) that  having students encounter vocabulary words often, and in various ways, can have a  significant effect on the development of increased reading vocabulary (…) | RCT (cluster) | Analyses of covariance (ANCOVAs) | Controlling for pretest vocabulary scores, the ANCOVA for the Stanford 9 Vocabulary Test revealed that students in the experimental group (*M* = 24.01; *SD* = 5.09) had significantly higher scores on the posttest than the students in the  control group (*M* = 22.99; *SD* = 6.07), *F*(1, 630) = 16.37, *p* < .001. | *F*(1, 630) = 16.37, *p* < .001. |  |
| *Brinchman et al., 2015* | The purpose of this study was to explore the hypothesis that teaching students  knowledge of word forms and meanings supports the development of  decoding and linguistic comprehension, which are fundamental components  of reading comprehension. | QE (treatment and control matched on grade and reading comprehension level) | Analyses of covariance (ANCOVAs). Group differences on the outcome measures at posttest were assessed using dummy coding of groups in a regression model, controlling for the corresponding pretest  measures and propensity scores as covariates. | (…) the effect on a measure of sentence formulation was also substantial (Cohen’s *d* = 0.76, *t* = 2.14, *p* = .032). The effect on affix knowledge was moderate in size (Cohen’s *d* = 0.55, *t* = 3.12, *p* = .001), and  the effect on a measure of reading comprehension was somewhat smaller (Cohen’s *d* = 0.30, *t* = 2.09, *p* = .039). | Grammar (sentence formulation):  Cohen’s *d* = 0.76, *t* = 2.14, *p* = .032  z = 2.145^a^ | Reading comprehension:  Cohen’s *d* = 0.30, *t* = 2.09, *p* = .039  z = 2.064^a^ |
| *Cable, 2007* | The purpose of this study is to expand the research base on effective instruction  for children with poor oral narrative ability by comparing the relative effects of a small  group, eight-week narrative intervention to a comparison condition by examining oral  narrative outcomes for second graders with weaknesses in narrative ability. | RCT (individual level, redistribution of Limited English Proficient students) | Analyses of covariance (ANCOVA), using the pretest of each  dependent variable, were performed for each dependent measure | The primary measure used to assess oral narrative ability was the oral narration  subtest of the TNL. The ANCOVA revealed a significant different between posttest  scores of the treatment and comparison group, *F*(2, 33) = 19.25, p < .001.  (no significant difference on narrative comprehension, *F*(2, 33) =  1.508, p = .228). | *F*(2, 33) = 19.25, p < .001 |  |
| *Clarke et al., 2010* | (…) There is therefore good reason to believe that an oral language  program that includes vocabulary training should lead to improvements in reading comprehension. | RCT RCT individual randomization within schools | Regression models with group dummy-coded and performance on the same measure at Time 1 and gender entered as covariates | (…) at both Times 3 and  4, all three intervention groups made significant gains relative to the control group on the WIAT II measure of reading comprehension. The bottom panel of Figure 2 shows the gains of the intervention groups relative to the control group on the NARA II measure of reading comprehension. The gains here are smaller than for the WIAT, and at Time 3, none of the intervention groups made significant gains relative to the control group. | WIAT II: OL group time 3: d = .69, p < .01.  z = 2.576 ^b^ | WIAT II: OL group time 4: d = 1.24, p < .01.  NARA II, OL group time 4: d = .45, p < .05. |
| *Coyne et al., 2010* | The purpose of this quasi-experimental study was  to evaluate the impact of an 18-week program of direct vocabulary instruction  in kindergarten on both proximal measures of target word knowledge as well  as transfer measures of overall vocabulary knowledge and listening comprehension. | QE | Multiple  regression. For each dependent variable, we estimated a  model that included the treatment condition, the centered initial PPVT score,  and the Treatment × PPVT interaction | The treatment group scored higher than the control group on the posttest administration of the PPVT (corrected *p* = .07) and there was an interaction between the fall PPVT score and treatment (corrected *p* = .08).  Although the estimated  treatment effect for spring PPVT was not statistically significant at the .05 level, the overall effect size of 0.60 can be considered substantively important.  Overall, effects trended in favor of the treatment group (corrected *p* = .11) and there was an interaction between pretest PPVT scores and the treatment (corrected *p* = .05). Although the estimated treatment effect for spring PPVT was not statistically significant at the .05 level, the overall effect size of 0.42 can be considered substantively important. | Excluded due to no significant effects on generalized measures |  |
| *Crain-Thoreson & Dale, 1999* | Our third research goal was to examine the effectiveness of adult instruction in Dialogic Reading in increasing  children’s vocabulary knowledge as measured by standardized tests (PPVT-R, EOWPVT-R). | RCT (individuals matched on receptive vocabulary in triads and randomly assigned to one of three groups) | A series of 2 x 3 repeated-measures analyses of variance with time (Pretest, Posttest) as a withinsubjects  factor and group membership (Parent, Staff/  practice, Staff/control) as a between-subjects factor | No statistically significant  effects of group membership or of time. | Excluded due to no significant effects on generalized measures |  |
| *Dockrell et al., 2010* | We predicted that when retesting the children: (1) the Talking Time intervention  would differentially improve children’s language skills in comparison with the two other  conditions; (2) the Story Reading intervention would also produce more improvement  in children’s language skills than the Non-intervention group | QE | Univariate ANCOVA with three levels of the  between-subjects factor group (Talking Time, Story Reading, and Non-intervention) with pretest scores as covariate | Significant differences between groups were found on three of the targeted language  measures (Verbal Comprehension, F(2, 95) = 3.32, p = .04; Naming  Vocabulary, F(2, 95) = 5.28, p = .007; Sentence Repetition F(2, 95) = 7.59, p = .001. | F(2, 95) = 3.32, p = .04 | F(2, 95) = 5.28, p = .007  F(2, 95) = 7.59, p = .001 |
| *Farver et al., 2009* | All of the activities in the curriculum have been  demonstrated in research to promote the development  of emergent literacy skills more quickly than  typical preschool activities | RCT (individual within blocks) | ANCOVAs were conducted  to compare scores on the five preliteracy skills in each language by group (control, English-only, and transitional) using both age and the Time 1 scores on the same measure as covariates. | There were significant  differences among the groups for children’s scores  on the English language skill assessments for  Receptive Vocabulary, F(2, 93) = 7.21, p = .001; Definitional  Vocabulary, F(2, 93) = 13.96, p < .001 | F(2, 93) = 7.21, p = .001 | F(2, 93) = 13.96, p < .001 |
| *Fricke et al., 2013* | We predicted that children  receiving the intervention programme would outperform  an untreated control group on measures of  language immediately after the intervention and  there would be transfer to literacy skills, | RCT (individual level) | Hierarchical linear models or structural equation  models (SEM) | At immediate and delayed posttest, there were  significant effects of the intervention on Language  (immediate posttest d = .80, z = 6.57, p < .001; maintenance  test d = .83, z = 2.41, p < .001), Narrative  (immediate posttest d = .39, z = 2.97; p = .003  maintenance test d = .30, z = 2.04, p = .041) | Language:  immediate posttest d = .80, z = 6.57, p < .001 | Lang.maintenance  test d = .83, z = 2.41, p < .001  Narrative:  immediate posttest d = .39, z = 2.97; p = .003  maintenance test d = .30, z = 2.04, p = .041 |
| *Fricke et al., 2017* | We had the following  hypotheses:  1. The intervention would lead to gains in oral language skills for children with poor language.  (…)  4. Since oral language interventions have been  found to promote reading comprehension (Clarke  et al., 2010; Fricke et al., 2013), we predicted  that the intervention group would show improved  reading comprehension. | RCT individual level | Structural equation models (SEM) were constructed  using Mplus 7.4  with Full Information Maximum Likelihood  estimators to allow for missing data and robust  (Huber–White) standard errors to allow for the clustering  of children within schools. | (…) both the 20-week and 30-week intervention groups show a significantly greater increase in their scores on the language posttest and delayed follow-up factors (controlling for pretest scores) than the waiting control group [d = .21 (95% CI 0.044–  0.366) and d = .30 (95% CI 0.130–0.468) respectively]. | 20-week: d = .21 (95% CI 0.044–  0.366)  z = 2.667 | 30-week: d = .30 (95% CI 0.130–0.468)  z = 3.917 |
| *Gonzales et al., 2010* | What is the effect of a science- and social-studies-focused shared book reading intervention  on standardized measures of receptive and expressive vocabulary? | RCT (cluster) | Multilevel modeling | On the receptive vocabulary measure,  children in the treatment group scored higher at posttest than did children in the comparison  group, PPVT–III (*γ* 01 = 7.57, *p* = .029, *δ*T = 0.93) after controlling for covariates. | *γ* 01 = 7.57, *p* = .029, *δ*T = 0.93  z = 2.184^a^ |  |
| *Hagen et al., 2017* | Our primary goal was to examine whether the intervention produced improvements on  a broad language factor that did not contain taught words. | RCT (cluster) | Structural equation  models (SEM) | Clear improvements from the intervention on this latent language  factor *d* = .563 [95% CI .280–.846] at the immediate posttest and *d* = .340 [95% CI .089–.591]  at the seven-month follow-up | *d* = .563 [95% CI .280–.846] at the immediate posttest  z = 3.9 | and *d* = .340 [95% CI .089–.591]  at the seven-month follow-up  z = 2.655 |
| *Haley et al., 2017* | We hypothesized that children who took part in the nursery intervention would perform better than the control group on measures of language  (both taught and standardized) immediately after the intervention programme. | RCT (individual) | Data were analysed using analyses of covariance  (ANCOVA) controlling for differences in gender, age  and baseline performance on each task | The intervention group perform significantly better  than the waiting control group only on the taught vocabulary  measures. | Excluded due to no significant effects on generalized measures |  |
| *Johanson & Arthur, 2016* | (…) Let’s Know!,  designed to enhance three component language skills (vocabulary, comprehension monitoring,  and text-structure knowledge) and overall language comprehension in prekindergarteners. | RCT (cluster, not wholly random selection) | Analysis of covariance using children’s pre-test scores as a covariate in a regression predicting  posttest scores on the LCM. | After adjusting for pre-test scores on the  LCM, children whose teachers implemented the Deep version had significantly greater  posttest LCM scores as compared to children in the BAU condition, with a large effect  size (B = 1.963, p < 0.0001, d = 0.63). | B = 1.963, p < 0.0001, d = 0.63  z = 3.719 ^b^ |  |
| *Justice et al., 2008* | The curriculum under investigation—the LFC—  was designed to improve children’s expressive language  abilities (…)primarily their use of complex syntax and diverse  vocabulary as measured within spontaneous language  samples. | RCT (cluster) | HLM | We found no main effects for LFC participation on  children’s expressive language skills | Excluded due to no significant effects on generalized measures |  |
| *Justice et al., 2010* | We hypothesized that children who were enrolled in classrooms  in which teachers implemented RIA would exhibit better  language and literacy skills at the end of the academic year  relative to children in classrooms in which teachers maintained  business-as-usual instruction. | QE | Hierarchical linear modeling (HLM;  Raudenbush & Bryk, 2002) was employed, accounting for nonindependence  and allowing examination of both child- and classroom level  components. | Children who participated  in the supplemental RIA curriculum demonstrated significantly higher  spring language performance than those in the comparison condition  in all areas: grammar (outscoring by 2.08 points, p = .02, effect  size = .24); morphology (3.20 points, p < .01, effect size = .35); and  vocabulary (2.68 points, p = .04, effect size = .17) | grammar (outscoring by 2.08 points, p = .02, effect  size = .24);  z = 2.327 ^a^ | morphology (3.20 points, p < .01, effect size = .35);  and  vocabulary (2.68 points, p = .04, effect size = .17) |
| *Kelley et al., 2015* | We hypothesized that preschool participants would demon-strate increases in both vocabulary knowledge and question-answering skills. | RCT (individual within blocks, N=18, 6 students x 3 classrooms) | A mixed 2 × 2 analysis of variance was conducted with one between-subjects factor (group) and one repeated-measure factor | On the PPVT-IV, there was no significant effect for group, F(1,16) = 0.12, p = 0.74. There was a significant main effect of time, F(1,16) = 34.21, p ≤ 0.001, __2= 0.68, but no significant group × timeinteraction, F(1, 17) = 0.28, p = 0.60. At posttest, the effect size ofthe difference between the treatment and comparison groups wassmall (d = 0.22). On the CELF-P2, there was no significant effectfor group, F(1, 16) = 0.78, p = 0.39, or time, F(1, 16) = 3.07, p = 0.10,and no significant treatment-by-time interaction, F(1, 16) = 0.53,p = 0.48. | Excluded due to no significant effects on generalized measures |  |
| *Van Kleeck et al., 2006* | We predicted that our book-reading intervention would replicate previous research that used dialogic reading to improve children’s literal language abilities (…). Our study was designed to further ask whether the intervention would also improve the inferential language skills of preschool children with language impairments. | RCT (individual) | A repeated measures analysis of variance (ANOVA) or analysis of covariance (Ancova) | PPVT-III: Results revealed a significant Group x Time interaction, F(1, 28) = 6.82, p = .01.  PLAI I & II: Results revealed a significant Group x Time interaction, F(1, 28) = 5.33, p = .03.  PLAI III & IV: Results revealed a significant group interaction, F(1, 28) = 5.54, p = .03. | F(1, 28) = 6.82, p = .01 | F(1, 28) = 5.33, p = .03.  F(1, 28) = 5.54, p = .03. |
| *Lawrence et al., 2015* | (...) improving reading comprehension by supporting students’ academic  vocabulary skills. | RCT (cluster) | HLM | (…) but no effects on a standardized assessment of general vocabulary | Excluded due to no significant effects on generalized measures |  |
| *Lawrence et al., 2017* | The goal of this study was to test the impacts of a brief discussion-based, vocabulary-focused  intervention on students’ knowledge of taught vocabulary, general vocabulary, and reading  comprehension. | RCT (cluster) | HLM | Though there was no  main treatment effect on the standardized measures of students’ general vocabulary knowledge or reading comprehension, baselineby-treatment interactions at the school and student level acted to attenuate the Matthew Effect in reading and vocabulary growth. | Excluded due to no significant effects on generalized measures |  |
| *Lesaux et al., 2014* | What is the impact  of an academic vocabulary program on the vocabulary knowledge, morphological  skills, reading comprehension, and writing skills of LM students and  their EO classmates enrolled in urban middle schools? | RCT (cluster) | Multilevel modeling (aka hierarchical linear modeling) | The main effect of treatment  was significant for a measure of (…)  and morphological derivation (d =  0.21, p< .0001)  (…) not significant for the standardized measures of reading comprehension  (d = 0.04, p = .4256) | d = 0.21, p< .0001  z = 3.719 ^b^ |  |
| *Lesaux et al., 2010* | What is the impact of an academic vocabulary  program on the vocabulary and reading comprehension  of language minority learners and their  native English speaker classmates enrolled in urban  middle schools? | QE | A sequence of multilevel  models in which the posttest score for each measure  was regressed on a dummy variable representing  condition (treatment or control) | Regarding the standardized  measures, the effect of treatment on Gates–  MacGinitie Reading Comprehension was relatively  small (*d* = 0.15; *p* = .0568), whereas the effect of treatment  on SAT-10 Reading Vocabulary was very small and  nonsignificant (*d* = 0.005; *p* = .5045). | Excluded due to no significant effects on generalized measures |  |
| *Lonigan et al., 1999* | In addition to examining the effects of  the interventions on children's oral language,  we also examined the effects on phonological  sensitivity and listening comprehension. (…)These three tests  were used to provide continuity with previous  research and to assess the domain of vocabulary  and expressive skills that the dialogic  reading program is intended to affect. | RCT (individual randomization within blocks) | A 3 (no-treatment group vs. typical reading  group vs. dialogic reading group) X 2 (pretest  vs. posttest) repeated measures analysis of covariance  (ANCOVA) was conducted on each  of the outcome measures using children's ages  at pretest and posttest as covariates. | The ANCOVA on the ITPA-VE revealed a  statistically significant group by time interaction,  *F* (2, 90) = 4.76, *p* = .01, but no overall  effect of time, *F* (1, 90) = 0.02, *p* = .90.  Planned comparisons revealed that changes in  ITPA-VE scores were greater for the combined  intervention groups than the no-treatment  group, *F* (1, 90) = 6.61, *p =* .01  The ANCOVA  for the WJ-LC using age and EOWPVT-R  scores as covariates revealed an effect of time,  *F* (1, 39) = 18.51, *p <* .001, and a group by  time interaction, *F* (1, 39) = 3.39, *p =* .04.  Planned comparisons indicated that changes in  WJ-LC scores across time were greater for the  combined intervention groups than the notreatment  group, *F* (1, 39) = 4.67, *p =* .04 | ITPA-VE: *F* (2, 90) = 4.76, *p* = .01 | ITPA-VE with the two interventions groups combined: *F* (1, 90) = 6.61, *p =* .01  Listening comprehension:  *F* (1, 39) = 3.39, *p =* .04.  Combined intervention groups: *F* (1, 39) = 4.67, *p =* .04 |
| *Lonigan et al., 2013* | We expected that each intervention would  result in significant gains in the skill it was intended to affect (e.g., children exposed to a  dialogic reading intervention would gain more vocabulary skills than children exposed to  simple shared reading or no shared reading) | RCT (individual randomization within blocks) | Multilevel modeling | Children who were in one of the groups that received the dialogic reading intervention  scored significantly higher than children in the groups that did not receive the dialogic  reading intervention on the Vocabulary composite, the EOWPVT-R, and the Basic Concepts  subtest of the CELF-P. | EOWPVT-R G1-G4: d = .25, p = .0255 (one-tailed) – result excluded due to non-significant result when two-tailed significanse test is used  EOWPVT-R G1-G5: d = .31, p = .009  z = 2.366^a^ | CELF-P: G1-G4: d = .31, p = .007  CELF-P: G1-G5: d = .31, p = .0095 |
| *Lonigan & Whitehurst, 1998* | We hypothesized that children's oral language skills would be impacted  positively in all three intervention groups compared to the control group. | RCT (individual randomization within blocks). Re-assigning needed. | A 4 (group) x 2 (center compliance) analysis of covariance  (ANCOVA) was conducted on each of the three posttest measures | The ANCOVA on the EOWPVT at posttest revealed a significant effect of intervention  group, F(3, 79) = 3.13, p = .03  The ANCOVA on the ITPA-VE at posttest revealed a significant effect of intervention  group, F(3, 79) = 4.94, p = .003 | F(3, 79) = 3.13, p = .03 | F(3, 79) = 4.94, p = .003 |
| *Murphy et al., 2016* | (…) to evaluate the effectiveness of a vocabulary intervention programme which focuses on multiple vocabulary learning strategies through whole class instruction, delivered by teachers  with support and training from a speech and language therapist, in mainstream schools  from areas of SED | QE | ANCOVA for between groups, taking account of age and T1 scores as covariates. | Only WC-E and BPVS-3 illustrated a significant time  by group interaction (WC-E = F(1, 199) = 4.016, *p* = 0.046, ηp  2 = 0.020; BPVS-3 = *F*(1, 198) = 5.419,  *p* = 0.021, ηp  2 = 0.027), where the students who had received the intervention significantly improved  from T1 to T2 compared to the students who had not received the intervention. | F(1, 199) = 4.016, *p* = 0.046 | *F*(1, 198) = 5.419,  *p* = 0.021 |
| *Neuman et al., 2011* | The primary goal of the present study was to examine the effects of WOW, based on the instructional principles delineated previously, for use in preschools with high numbers of economically disadvantaged learners to bolster their vocabulary and conceptual development. | RCT (cluster) | HLM and ANCOVA | However, there was no significant difference between groups in scores on the Woodcock-Johnson Picture Vocabulary Subtest. | Excluded due to no significant effects on generalized measures |  |
| *Nielsen & Friesen, 2012* | Can a small-group model of instruction delivered over 12 weeks affect at-risk kindergarten students’ knowledge of taught vocabulary  words and their ability to retell stories? | QE |  | There were no statistically significant differences between  groups on the TOLD semantic composite posttest.  There were no statistically significant differences between groups  on the posttest of the Test of Narrative Language (TNL) | Excluded due to no significant effects on generalized measures |  |
| *Phillips et al., 2016* | (…) the goal for this study was to demonstrate efficacy in a rigorous, well-controlled randomized  trial. (…) The hypotheses were that treated children would outperform the untreated children, particularly on aligned, proximal  measures of syntax and listening comprehension. | RCT individual | SPSS mixed models that treated preschool  center as a fixed factor were conducted using restricted  maximum likelihood estimation and raw scores on all measures. | Although effects  on distal, standardized language measures did not  reach significance given the modest sample size, the significant  effects on our Listening Comprehension measures  and the marginally significant effect size for the OWLS:LC  suggests potential for affecting oral comprehension. | Excluded due to no significant effects on generalized measures |  |
| *Pollard-Durodola et al., 2011* | We specifically examined the effect of this intervention  on the receptive and expressive vocabulary  of preschool students at risk for vocabulary  delay primarily associated with low SES. | RCT (cluster) | Analysis of covariance (ANCOVA)  model, with the pretest measures and demographic  variables (i.e., gender, age, English learner status, ethnicity, and special education status)  as covariates. | The results of the analysis indicated  no statistically significant main effects of condition  on the PPVT-III (*γ* = 0.52, *p* = .802) or on  the EOWPVT (*γ* = 0.64, *p* = .701) | Excluded due to no significant effects on generalized measures |  |
| *Proctor et al., 2011* | Specifically, we sought to test the effects of working in an internet-based SDR on  standardized and researcher-designed measures of vocabulary and comprehension. | QE | Repeated  measures ANOVAs | ANOVAs were run on the Gates Vocabulary and Comprehension measures, with a  significant effect for vocabulary growth among all participants (F[1, 214] = 57.2,  p<.001), with no effect for condition (F[1,214] = 1.3, p = .26) or its interaction  with time (F<1). Similar results were obtained for Gates Comprehension, with  significant growth for all students (F[1, 211] = 36.0, p<.001), no condition effect  (F[1,211] = 1.3, p = .26) or its interaction with time (F<1). | Excluded due to no significant effects on generalized measures |  |
| *Rogde et al., 2016* | We hypothesized that the general language program would produce improvements  in the intervention group on both custom and standardized outcome measures  of language proficiency. | RCT (individual) | Latent autoregressive models (structural equation models) | There was a clear positive effect of the  intervention immediately after the intervention (posttest 1) (d = .55, p = . 000). This effect was  still present at the follow-up sevenmonths later (posttest 2) (d = .26, p = .037).  No effect of the  intervention was found for receptive language | Posttest1: d = .55, p = . 000  z = 4.237 | Posttest2: d = .26, p = .037  z = 2.086 |
| *Schaefer et al., 2016* | The 18-week intervention programme was designed  to help improve children’s active listening, vocabulary, language comprehension, and  narrative skills and support the development of phonological awareness | RCT (individual) | Latent variable models in Mplus | No generalisation effects were found on any primary or secondary outcomes (including  word level literacy) apart from a small improvement in narrative comprehension at posttest (*d* =  0.35). | *p* = .022, *d* = .35  z = 2.29^a^ |  |
| *Silverman et al., 2013* | The goals of the present study were to explore the effect of a read aloud plus extension activities  intervention relative to an intervention that included read alouds alone. | RCT (cluster) | Hierarchical linear modeling | Neither intervention had an effect on general word knowledge. | Excluded due to no significant effects on generalized measures |  |
| *Simmons et al., 2010* | What are the effects of multiple-strategy interventions (i.e., cognitive comprehension  and content vocabulary) compared to typical social studies practice  on fourth-grade students’ performance on general and social studies  vocabulary and reading comprehension assessments? | RCT (cluster) | Structural equation  modeling | No reliable differences  between typical practice and either experimental intervention were observed  on the general standardized measures of reading comprehension (GMRT-4)  or social studies vocabulary (TORC-3 SS). | Excluded due to no significant effects on generalized measures |  |
| *Spencer et al., 2014* | First, we conducted an efficacy study to investigate the effect of narrative intervention, delivered in a large group arrangement (i.e. whole class), on diverse preschoolers’ narrative language skills. | QE | ANCOVA | Story retell posttest: The ANCOVA was significant, *F*(1, 68) = 4.11, mean squared error (*MSE*) = 129.69, *p* = .046, *d* = 0.49. follow-up: *F*(1, 68) = 4.45, *MSE* = 146.93, *p* = .039, *d* = 0.51.  Story comprehension posttest: The ANCOVA was significant, *F*(1, 68) = 5.41, *MSE* = 39.88, *p* = .023, *d* = 0.56. Follow-up: The simple main effects test was significant for a low pretest story comprehension score, *F*(1, 67) = 27.33, *p* < .0001, *d* = 1.26, and for a medium value on the covariate, *F*(1, 67) = 26.55, *p* < .0001, *d* = 1.24. | *F*(1, 68) = 4.11, *p* = .046 | *F*(1, 68) = 4.45, *p* = .039.  *F*(1, 68) = 5.41, *p* = .023.  *F*(1, 67) = 27.33, *p* < .0001.  *F*(1, 67) = 26.55, *p* < .0001. |
| *Styles & Bradshaw, 2015* | What is the impact of the speaking and listening intervention on reading ability? | RCT (individual within blocks) | ANOVA | An ANOVA of the NGRT posttest reading score by randomized group did show a significant impact of the intervention (F = 6.41, p = 0.01, n = 221) but the CELF score did not show a significant impact (…). | F(1, 220) = 6.41, p = 0.01, n = 221 |  |
| *Vadasy et al., 2015* | A multi-cohort cluster randomized trial was conducted to estimate effects of rich vocabulary  classroom instruction on vocabulary and reading comprehension. | RCT (cluster) | Three-level hierarchical linear modeling | Results for both vocabulary and comprehension outcomes (shown in Table 8) showed that,  adjusted for student and classroom pretest levels, students in RVOC treatment classrooms  outperformed their peers in control classrooms on all measures. For distal measures,  the adjusted group mean differences were estimated at 0.46 and 0.64 points for vocabulary  and comprehension, respectively. | ITBS-Vocab:  Coeff. = 26.90, *p <* .001  z = 3.09 ^b^ | ITBS-Compreh:  Coeff. = 32.19, *p <* .001 |
| *Valdez-Menchca & Whitehurst, 1992* | The present study extends the techniques of shared picture  book reading used by Whitehurst et al. (1988) to children of  low-income parents attending public day care in Mexico | QE (A matched-pair experimental design) | We assessed the effects of the intervention  on the standardized tests by conducting separate two-group  (experimental vs. control), two-tailed *t* tests on children's standard  scores. | Each of these analyses revealed a significant group  effect, indicating a higher performance by children in the experimental  group than by children in the control group, *t*(18) =  2.57, *p* = .019, for PPVT-R; *t*(18) = 3.06, *p* = .007, for  EOWPVT; and *t*(18) = 3.38, *p =* .003, for ITPA. | PPVT-R:  *t*(18) = 2.57, *p* = .019 | EOWPVT:  *t*(18) = 3.06, *p* = .007.  ITPA:  *t*(18) = 3.38, *p =* .003. |
| *Wasik & Bond, 2001* | (…)because teachers were trained to use book reading strategies  that focused on increasing the use of language and emphasizing  vocabulary, it was hypothesized that this intervention would  have a more broad effect on children's vocabulary development. | RCT cluster (Two teachers were randomly assigned to the intervention condition, and two were assigned to the control condition.) | ANOVA  comparing treatment and control classrooms for just the A.M.  classes and an ANOVA comparing treatment and control classrooms  for just the P.M. classes. | The analyses with classroom as the unit of analysis revealed the expected interaction for both the A.M. classes, F(l, 2) = 62.73, *p <* .016, and P.M. classes, F(l, 2) = 346.08, *p <* .001. The comparable analyses with students as the unit of analysis produced the same Treatment X Trial interaction, F(l,120) = 13.69, *p <* .001. | A.M. classes:  *F*(l, 2) = 62.73, *p <* .016.  z = 2.145^c^ | P.M. classes:  *F*(l, 2) = 346.08, *p <* .001.  Students as unit of analysis: *F*(l,120) = 13.69, *p <* .001. |
| *Whitehurst et al., 1994* | We hypothesized  that children who were read to by teachers in day care would show increments in language ability compared with a control group and that children who were read to by teachers and parents would show even stronger effects, | RCT (individual within blocks) | A 5 (day-care centers) X 3 (treatment conditions) analysis of  covariance (ANCOVA) was conducted for each of the four posttests  and each of the three follow-up tests | The ANCOVA on One Word scores at posttest (with three covariates) produced significant effects for center, *F(2,* 49) = 3.77, *p =* .009, and condition, *F(2,*49) = 6.84, *p =* .002, but not the interaction, F(8, 49) = 1.55, *p* = . 164. Follow-up: a significant effect for condition, *F(2,* 34) = 3.62, *p =* .037.  An ANCOVA on PPVT-R posttest scores (with two covariates) produced a marginal effect for the interaction of center and condition, F(8, 50) = 1.95, *p =* .073, and no effects for  center, F(4, 50) = 0.18, *p =* .948, or condition, *F(2,* 50) = 1.22, *p =* .303. An ANCOVA on the PPVT-R follow-up scores (with  one covariate) produced a significant interaction effect, F(8,37) = 2.22, *p =* .048, but no effects for center, F(4, 37) = 0.77, *p =*  .549, or condition, *F(2,* 37) = 0.80, *p =* .459.  An ANCOVA on the Our Word at posttest (with two covariates) produced significant effects for both center, F(4, 50) = 2.89, *p =* .031, and condition, F(2, 50) = 3.46, *p =* .039, but not for the interaction, F(8, 50) = 1.25, *p* = .292. | *F*(2,49) = 6.84, *p =* .002 | *F*(2, 34) = 3.62, *p =* .037  *F*(8,37) = 2.22, *p =* .048  *F*(4, 50) = 2.89, *p =* .031  *F*(2, 50) = 3.46, *p =* .039 |

^a^z-score calculated from exact p-value, ^b^ z-score (one-tailed) was calculated based on p as if p was precise (e.g. p < 0.01 treated as p =0 .01),^c^ z-score (one-tailed) was calculated based on p = 0.016 because reported results produced syntax error.

# Online supplement 5: Forest plots, stratified analyses on differential language outcomes

## Vocabulary (overall)

Immediate effects on vocabulary outcomes

Long-term effects on vocabulary outcomes

## Vocabulary reading

Immediate effects on vocabulary reading outcomes

## Vocabulary expressive

Immediate effects on vocabulary expressive outcomes

Long-term effects on vocabulary expressive outcomes

## Vocabulary receptive

Immediate effects on receptive vocabulary outcomes

## Grammar

Immediate effects on grammar outcomes

## Narrative and listening comprehension

Immediate effects on narrative and listening comprehension outcomes

Long-term effects on narrative and listening comprehension outcomes
